# Photoelectrochemical CO_2_ Reduction at a Direct CuInGaS_2_/Electrolyte Junction

**DOI:** 10.1021/acsenergylett.3c00022

**Published:** 2023-03-02

**Authors:** Yongpeng Liu, Meng Xia, Dan Ren, Simon Nussbaum, Jun-Ho Yum, Michael Grätzel, Néstor Guijarro, Kevin Sivula

**Affiliations:** Institute of Chemical Sciences and Engineering, École Polytechnique Fédérale de Lausanne (EPFL), Station 6, 1015 Lausanne, Switzerland

## Abstract

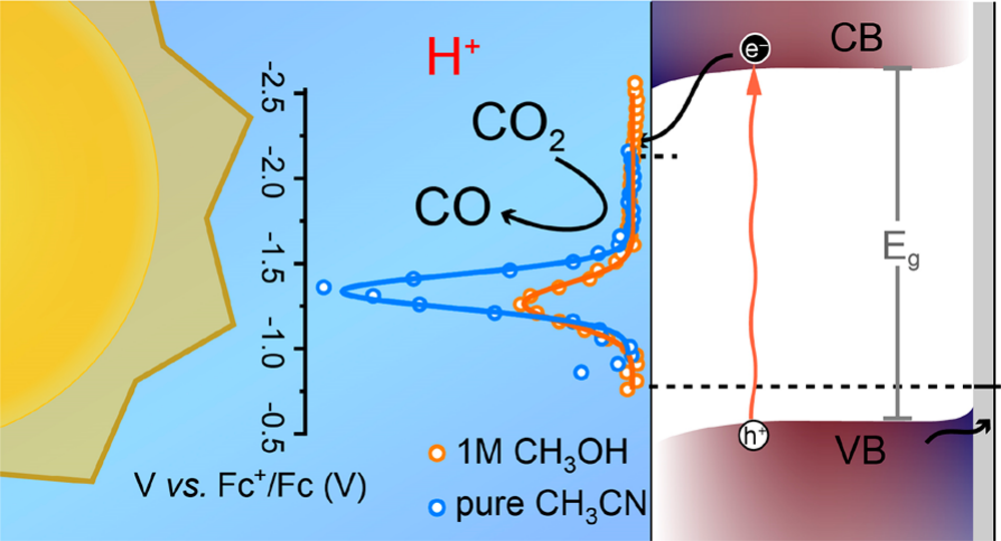

Photoelectrochemical (PEC) CO_2_ reduction has received considerable attention given the inherent sustainability and simplicity of directly converting solar energy into carbon-based chemical fuels. However, complex photocathode architectures with protecting layers and cocatalysts are typically needed for selective and stable operation. We report herein that bare CuIn_0.3_Ga_0.7_S_2_ photocathodes can drive the PEC CO_2_ reduction with a benchmarking 1 Sun photocurrent density of over 2 mA/cm^2^ (at −2 V vs Fc^+^/Fc) and a product selectivity of up to 87% for CO (CO/all products) production while also displaying long-term stability for syngas production (over 44 h). Importantly, spectroelectrochemical analysis using PEC impedance spectroscopy (PEIS) and intensity-modulated photocurrent spectroscopy (IMPS) complements PEC data to reveal that tailoring the proton donor ability of the electrolyte is crucial for enhancing the performance, selectivity, and durability of the photocathode. When a moderate amount of protons is present, the density of photogenerated charges accumulated at the interface drops significantly, suggesting a faster charge transfer process. However, with a high concentration of proton donors, the H_2_ evolution reaction is preferred.

Elucidating the interplay between the chemical environment, interfacial carrier dynamics, and catalysis is crucial to understand and optimize the chemical conversion at semiconductor-liquid junctions (SCLJs). Photoelectrochemical (PEC) CO_2_ reduction has been drawing increasing attention with the prospects of manufacturing a wide range of platform chemicals, such as CO, formic acid, or methane, among others, using CO_2_ as feedstock and sunlight to power the entire process.^1−3^ Recent advances in the field have relied on the incorporation of cocatalysts on the semiconductor surface.^4−6^ Here, semiconductor pnictides, metal oxides, and chalcogenides operate as light absorbers, transferring the highly reducing photogenerated electrons to cocatalysts wherein the electrochemical CO_2_ reduction reaction (CO_2_RR) takes place via a specific pathway to select the reduction product. Metalorganic complexes and enzymes have shown high product selectivity and remarkably low overpotentials.^7−9^ However, their relative high cost, the need for specific grafting steps to anchor the cocatalyst, and the concerns about the long-term stability at high current densities—especially for biocatalysts—raises doubts about their practicality for large-scale industrial application. Alternatively, inorganic cocatalysts such as transition and post-transition metals^10−12^—and more recently metal oxides and chalcogenides—have been demonstrated to activate the PEC CO_2_RR toward specific products.^13,14^ Given that the structure and composition of these catalysts resemble that of state-of-the-art semiconductors, it is reasonable to argue that PEC CO_2_ conversion could directly occur at SCLJs provided that the reactive interface mimics the characteristics of such catalytic phases.

Although scarce, a few examples of the PEC CO_2_RR occurring at SCLJs have been reported.^15^ For instance, Gu et al. reported a Faradaic efficiency of ca. 9% for PEC CO_2_-to-formate conversion using Mg-doped CuFeO_2_ photocathodes.^16^ The authors concluded that, despite the presence of metallic copper on the surface, which could contribute to the catalysis, the native oxide presumably governed the CO_2_-to-formate conversion. Similarly, chalcogenides such as CuGaS_2_ or Cu_2_ZnSnS_4_ can drive the photoreduction of CO_2_ yielding CO.^17,18^ While these preliminary studies imply the reactivity of the direct SCLJ, they still display relatively poor selectivity and current density (i.e., with 1 Sun photocurrent densities <1 mA cm^–2^). Therefore, gathering new insights on the reaction pathway and the kinetics of the PEC CO_2_RR directly occurring at the semiconductor’s surface holds the key to advance a new generation of cocatalyst-free photocathodes with improved performance. With this purpose, here, we present a comprehensive analysis of the PEC reactivity of a bare semiconductor photocathode as a function of the proton donor ability of the solvent to unravel the reaction mechanism while applying an assortment of spectroelectrochemical tools to characterize the electronic and kinetic features of the interface during the photoelectrosynthetic reaction.

Pristine CuIn_0.3_Ga_0.7_S_2_ (CIGS) was chosen as model material leveraging our previous demonstration of PEC water reduction^19,20^ and its elemental composition, which matches that of reported catalytic centers for CO_2_ reduction.^13,15,21^ A description of the fabrication of the CIGS photocathodes as well as a complete characterization of (i) the morphology by means of scanning electron microscopy (SEM, Figure S3) and atomic force microscopy (AFM, Figure S4), (ii) the elemental composition by using energy-dispersive X-ray spectroscopy (EDX, Figure S5), and (iii) the crystal structure by X-ray diffraction (XRD) and Raman microscope (Figure S6) can be found in the Supporting Information. The PEC activity of the bare CIGS photocathodes was evaluated by means of linear sweep voltammetry (LSV), under intermittent simulated AM 1.5G illumination (1 Sun) in either Ar- or CO_2_-saturated 0.1 M Bu_4_NPF_6_ solution (Figure 1). Note that anhydrous acetonitrile (ACN), 1 M CH_3_OH in ACN (M-ACN), and anhydrous methanol (AM) were tested as solvents. In all cases, the PEC response improves with the CO_2_ purging, although to a different extent depending on the solvent. Higher photocurrent density and more positive photocurrent onset were recorded, suggesting that the CO_2_ was photoelectrochemically reduced. It is worth noting that while in the case of ACN the improvement was apparent (Figure 1a), the incorporation of small amounts of methanol led to a drastic enhancement of the performance (Figure 1b). Indeed, the photocurrent density increased from 0.54 mA cm^–2^ (Ar) up to 2.18 mA cm^–2^ (CO_2_) at −2 V vs Fc^+^/Fc, while the turn-on voltage shifted around 400 mV toward more positive potentials. However, when AM was replaced as solvent, the changes recorded with CO_2_ were modest, mostly evidenced by a slight shift in the photocurrent onset rather than in the magnitude of the photocurrent (Figure 1c). Note that incident photon-to-current efficiency (IPCE) measurements were performed to confirm that the photocurrent recorded by LSV matches the integrated photocurrent considering the standard AM 1.5G spectrum (Figure S7). Likewise, differences in the solubility of CO_2_ were also considered as an origin for the changes in the photocurrent delivered. Since the solubility of CO_2_ in AM (0.16 M) is lower than in ACN (0.27 M),^22^ no significant changes in the concentration of CO_2_ in M-ACN are expected. We note the presence of dark current, ascribed to the HER and/or the CO_2_RR occurring at the exposed substrate (sulfurized Mo) or to corrosion (in the case of Ar-purged ACN). A slightly larger dark current in AM suggests the larger contribution of the HER in that case.

**Figure 1:**
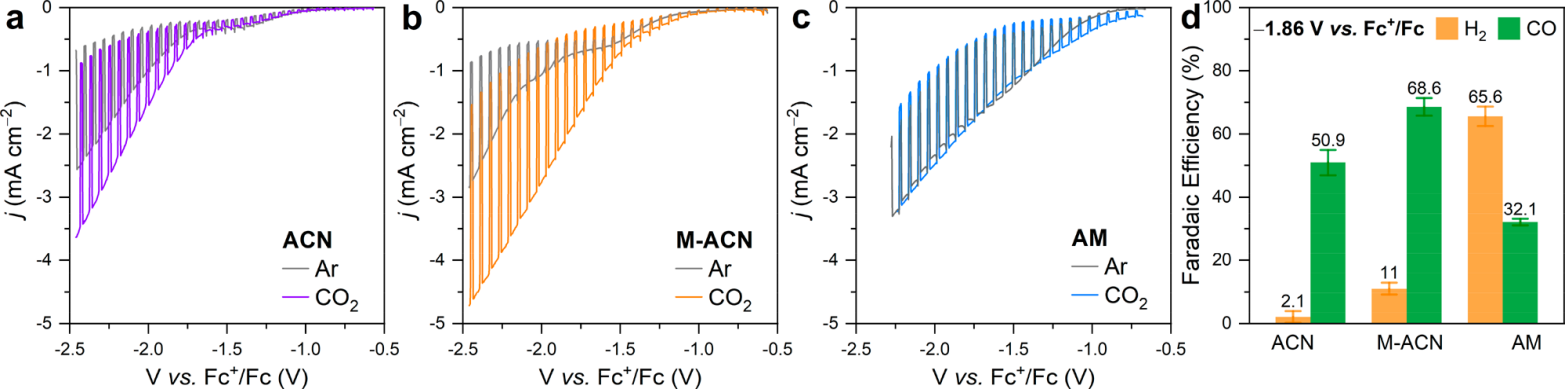
Current-voltage characteristic under intermittent AM 1.5G illumination of CIGS photocathodes measured in Ar- or CO_2_-saturated (a) ACN, (b) M-ACN, and (c) AM, with 0.1 M Bu_4_NPF_6_ as the supporting electrolyte. The scan rate was 20 mV/s toward more negative potentials. (d) Comparison of Faradaic efficiency recorded at −1.86 V vs Fc^+^/Fc in three different electrolytes. The error bars originated from measurements of three different samples.

To assess the selectivity of the PEC process, the gases produced (H_2_ and CO) under illumination and at −1.86 V vs Fc^+^/Fc were quantified as a function of the solvent composition by means of gas chromatography (Figure S2). We note that methanol is commonly used as a hole scavenger in photocatalytic transformations, giving rise to H_2_ as a byproduct. Thus, control experiments were performed to confirm that the measured H_2_ originates from the cathodic reaction instead of from the methanol oxidation (Figure S8, Table S1). Then, looking at the gas evolution results (Figure 1d) and based on the observed production of CO and H_2_, CO_2_ reduction was favored in ACN and M-ACN, whereas the reduction of protons dominated in AM. In fact, the Faradaic efficiency (FE) for the CO_2_-to-CO conversion increased from 50.9 ± 4.0% (ACN) to up to 68.6 ± 2.8% (M-ACN, corresponding to a partial-photocurrent density for CO_2_-to-CO conversion, , of 1.4 mA cm^–2^) when methanol was added but dropped to 32.1 ± 4.0% in AM. Interestingly, these results not only confirm that PEC CO_2_ reduction can actively occur at the SCLJ but more importantly suggest that the selectivity and efficiency can be controlled by adjusting the proton availability. This latter observation is consistent with reports of (dark) electrochemical CO_2_ reduction, which has proven to be highly sensitive to electrolyte composition.^23,24^ Generally speaking, the CO_2_-to-CO conversion begins with the reductive (one-electron transfer) adsorption of CO_2_ onto the catalytic surface in the form of a CO_2_^•–^ anion radicals.^25,26^ In the presence of an aprotic solvent, this intermediate interacts with another CO_2_ molecule and accepts a second electron to release CO and carbonate. In the presence of a proton donor, however, protonation of the anion radical occurs followed by dehydroxylation and a second electron transfer to release CO.^27^ In dark electrocatalysis, this modification of the reaction pathway induces changes in the CO_2_-to-CO activity primarily ascribed to the additional stabilization that the protonation brings to the adsorbed intermediate.^27^ With this in mind, we speculate that the protonation of the intermediate on the SCLJ will not only decrease the photocathodic overpotential but also accelerate the rate of the reaction, since the reductive adsorption of CO_2_ is generally accepted to be the rate-determining step. While the improved  and onset potential for CO_2_-to-CO conversion in M-ACN are well rationalized in these terms, the deactivation of CO_2_ reduction in favor of the HER in AM requires another explanation. Bearing in mind that bare CIGS photocathodes have been shown to drive the HER in aqueous electrolyte (without CO_2_)^19^ and that the catalytic sites involved in the CO_2_ reduction are likely the same as those driving the HER,^13,19,28^ we hypothesize that, in the presence of a large proton donor concentration, most of the catalytic sites will be saturated with protons, leaving a small percentage of sites available for the CO_2_ reduction to take place. It is worth noting that in AM the gaseous product is syngas with a H_2_:CO ratio of 2:1, which is a desirable composition to feed industrial processes.^29^ To validate that CO originates from CO_2_ reduction instead of other sources such as degradation of the organic solvent, control experiments have been performed in N_2_-saturated electrolytes under PEC conditions. As shown in the GC traces in Figure S9, no sign of CO is observed in the absence of CO_2_, indicating that CO is produced by CO_2_ reduction.

Careful investigation of the relative production of CO and H_2_ reveals that, especially in ACN, the partial currents for H_2_ and CO do not account for the entire photocurrent density recorded experimentally. Indeed, about 47% of the photocurrent is not identified in ACN. Since no other gaseous (gas chromatography) or liquid (^1^H NMR) products were detected, it is plausible that part of the photocurrent is due to corrosion. This motivates the further investigation of the durability of the CIGS photocathodes. Operating under continuous CO_2_ photoreduction conditions, the photocurrent density versus time behavior was evaluated in each solvent condition by means of chronoamperometry. As shown in Figure 2a, in ACN, the photocurrent density recorded at −1.86 V vs Fc^+^/Fc dropped about 57% of its initial value after 2000 s. Given the mismatch between the detected products and the recorded photocurrent, photocorrosion is most likely responsible for the deterioration of the performance. The addition of a proton donor in M-ACN significantly lengthens the stability (Figure 2b), maintaining about 90% of the photocurrent density after 2000 s and 70% after 4000 s. We hypothesize that the accelerated degradation at longer times of operation arises from the evaporative loss of methanol during CO_2_ purging. However, additional experiments would be needed to verify this. Surprisingly, in AM, the stability is greatly enhanced (Figure 2c), virtually showing no degradation of the performance after 44 h of continuous operation. The correlation between the stability and the proton availability could be explained considering the kinetic competition existing between the photocorrosion phenomena and the electron transfer at the interface. In a protic solvent like AM, the fast HER depletes photogenerated electrons accumulated at the interface, while the CO_2_-to-CO conversion in a proton-rich environment will proceed preferentially via the aforementioned protonation of the intermediate—accelerating the transformation. Thus, photocorrosion is outcompeted by both the HER and CO_2_ reduction reaction. In contrast, in an aprotic environment such as ACN, the relative slowdown of the reductive adsorption of CO_2_ could allow the photocorrosion processes to take place at a considerable rate. Obviously, in the presence of M-ACN, it is reasonable to consider that both protonated and nonprotonated intermediates will coexist at the surface evolving to CO via different mechanisms, while photocorrosion processes will contribute to the photocurrent to a lesser extent.

**Figure 2:**
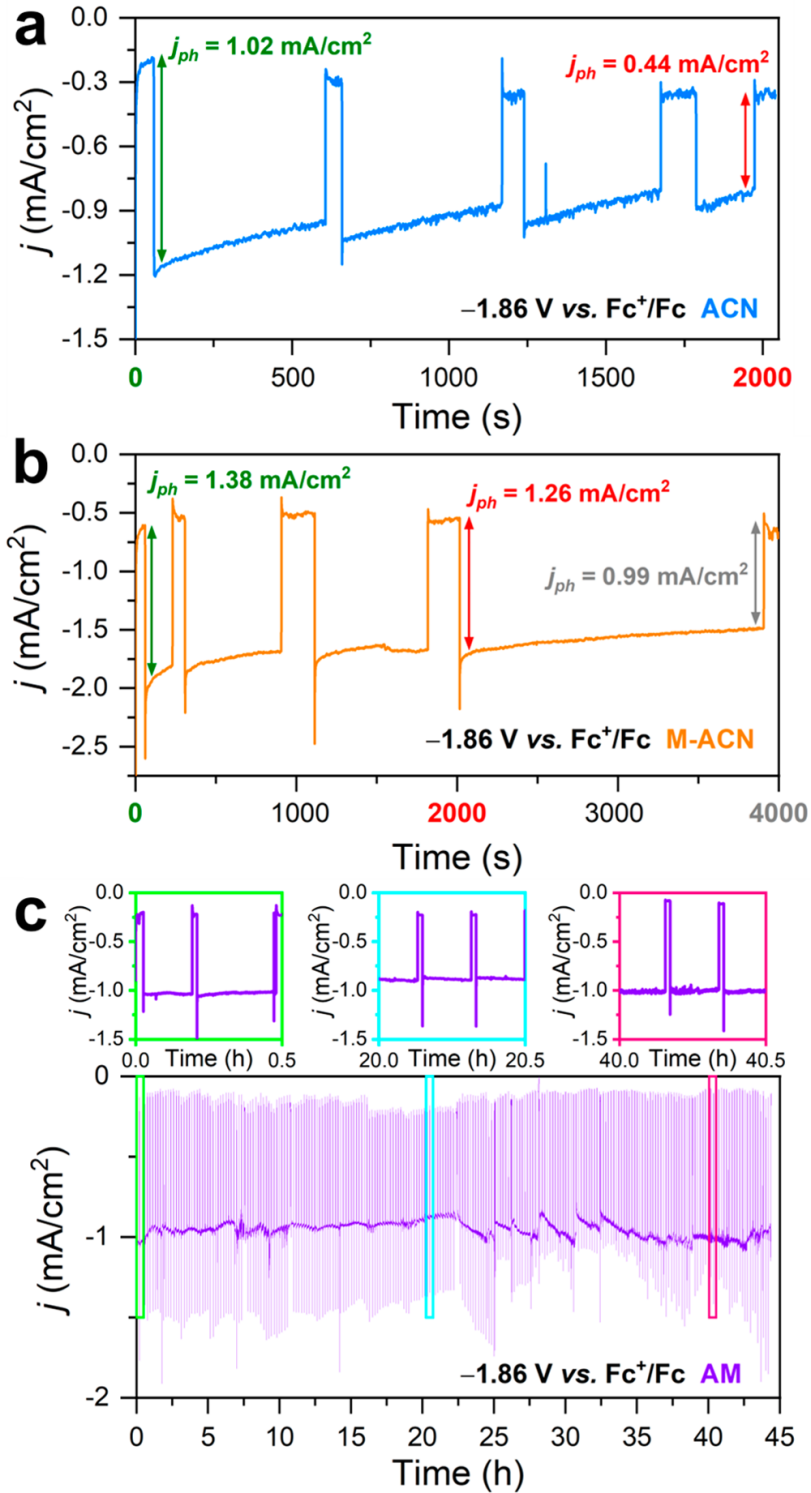
Stability tests via the chronoamperometry of CIGS photocathodes for photoelectrochemical CO_2_ reduction in CO_2_-saturated (a) ACN, (b) M-ACN, and (c) AM, with 0.1 M Bu_4_NPF_6_ as the supporting electrolyte.

Following the improved selectivity toward CO in M-ACN, we next explored the influence of the applied potential on the product distribution. Figure 3a displays the FE for CO and H_2_ production as a function of the applied potential from −1.46 V up to −1.86 V vs Fc^+^/Fc. Interestingly, at the most positive applied bias, H_2_ is preferentially formed. This could be accounted for by the higher overpotential for CO_2_ reduction. At more negative applied potentials, the FE for H_2_ production remains stable at around 10%, while the FE for CO increases up to 68.6%, which is among the highest reported for a pristine photocathode (Table S2). However, the fact that the FE for H_2_ and CO does not account for the total current density, especially at more positive potentials, could, again, be justified by the presence of photocorrosion processes. In fact, the more positive the applied potential, the lower (on an energy scale) the quasi-Fermi level of the photogenerated electrons. This reduces the driving force for electron transfer and favors photocorrosion. To better illustrate the relative production of CO, Figure 3b displays the percentage of CO produced with respect all the products. As shown, the relative selectivity toward CO increases rapidly from about 25% at −1.46 V to 87% saturating from −1.76 V vs Fc^+^/Fc onward. Interestingly, the switching of the product distribution occurs in a very narrow potential window, that is, between −1.46 and −1.56 V vs Fc^+^/Fc. This suggests that within this range the threshold potential to trigger the CO_2_-to-CO conversion has been surpassed, thus affording CO as the main product.

**Figure 3:**
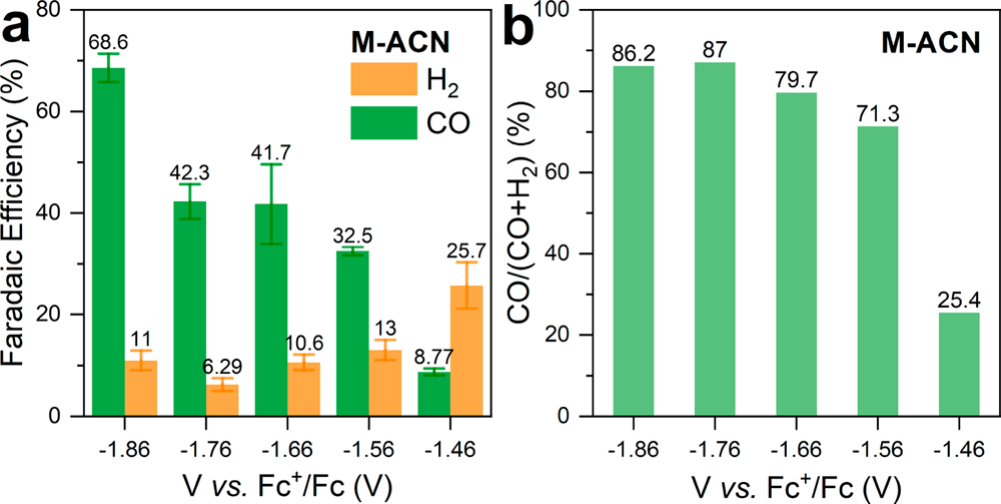
(a) Faradaic efficiency and (b) CO selectivity in M-ACN. The error bars originated from measurements of three different samples.

To gain a deeper understanding on how the proton availability impacts the electronic structure of the SCLJ, CIGS photocathodes were characterized by electrochemical impedance spectroscopy (EIS). A Mott–Schottky (MS) plot (Figure 4a) was constructed based on the capacitance values extracted from EIS measurements performed in the dark in M-ACN (Figure S10) and suggests that the flat band potential (*V*_*fb*_) is located at about −0.5 V vs Fc^+^/Fc, which is consistent with the *V*_*fb*_ estimated from the Butler plot (Figure S11). This is in stark contrast to the steep onset of photocurrent that occurs at a significantly more negative applied potential, i.e., not until −1.3 V vs Fc^+^/Fc (Figure 1b). Such sluggish photocurrent development has been previously attributed to the occurrence of Fermi level pinning (FLP) caused by surface trap states.^30^ Based on the *V*_*fb*_ and the band gap (estimated to be ca. 2.0 eV from the onset of the IPCE spectrum), a tentative energy diagram of the CIGS including relative position with respect the CO_2_/CO redox potential was constructed, to confirm that photogenerated electrons are reducing enough to drive the PEC CO_2_ reduction (Figure S12). Next, EIS was performed under illumination in both ACN and M-ACN. Figure 4a shows the density of surface states (DOSS) as a function of the applied potential. The DOSS was estimated from the Nyquist plots as described in the Supporting Information. Qualitatively, it is clear that the magnitude of the DOSS signal decreases with the addition of methanol. Using a Gaussian fit allows estimation of the total density of surface states (*N*_*SS*_), which decreased from 4.7 × 10^13^ to 2.1 × 10^13^ cm^–2^. Bearing in mind that the DOSS is a proxy for the concentration of charges accumulated at the CIGS surface,^19^ these results suggest that, under operation, the buildup of electrons at the SCLJ decreased by half with the addition of methanol. This is not unreasonable, since methanol is expected to accelerate the adsorption of the CO_2_, speeding up the CO_2_-to-CO conversion and ultimately stabilizing a lower surface electron concentration in steady-state conditions. Note that, in both solvents, the DOSS lies in the potential window from −0.9 up to −1.6 V vs Fc^+^/Fc, overlapping with the region where the photocurrent displays only a minor increase. This is a common feature of PEC systems applied for catalytic conversions, such as water reduction and oxidation, wherein surface charging (and the formation of the reaction intermediates) precedes the desired PEC conversion.^19,31^

**Figure 4:**
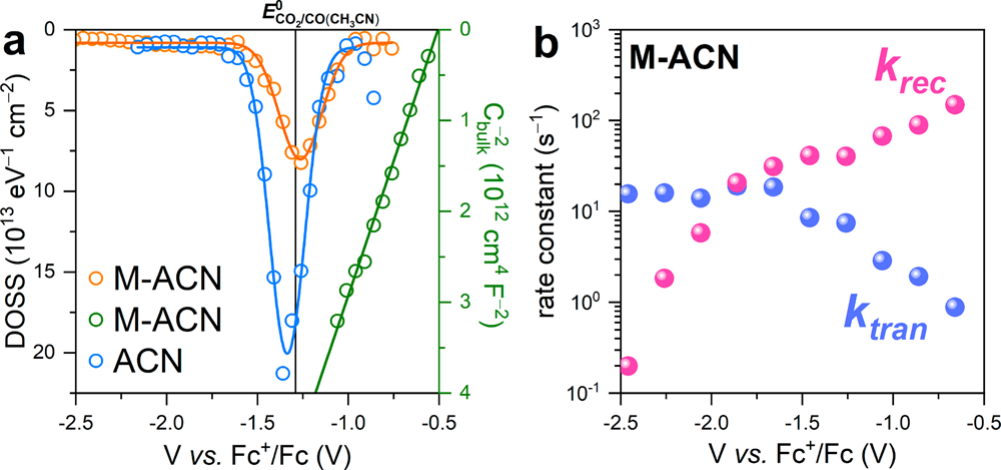
(a) Energetic distribution of density of surface states (DOSS, open circle) with corresponding Gaussian fit (solid line) in different electrolytes and MS plot (olive open circle) with linear regression (olive line) in M-ACN. The black vertical line indicates the reversible potential of the CO_2_/CO redox couple in ACN.^32^ (b) Pseudo-first-order rate constants for charge transfer *k*_*tran*_ and surface recombination *k*_*rec*_ as a function of applied potential in M-ACN.

Intensity-modulated photocurrent spectroscopy (IMPS) was next utilized to interrogate the charge carrier dynamics at the SCLJ during CO_2_ conversion. So far, only two reports have implemented IMPS to characterize CO_2_ reduction (i.e., on TiO_2_/CdS photocatalysts and CuBi_2_O_4_ photocathodes).^33,34^ While in these previous studies IMPS was merely used to extract a time constant, here, we expand this tool to determine the rate constants for charge transfer (*k*_*tran*_) and recombination (*k*_*rec*_) as a function of the applied potential in M-ACN (Figure 4b). A complete description of the model used to extract the pseudo-first-order rate constants and the corresponding Nyquist plots (Figure S13) as well as the charge transfer efficiency (Figure S14) can be found in the Supporting Information. As observed, the variation of *k*_*rec*_ with the applied potential displays two distinct slopes with a crossover potential at −1.5 V vs Fc^+^/Fc. At more positive applied potentials, the small variation of *k*_*rec*_ could be attributed to the existence of FLP in this region. For instance, the presence of surface traps in this potential window would cause the applied potential to drop at the Helmholtz layer, i.e., charging the interface, rather than at the space charge region, therefore leaving the band bending that controls *k*_*rec*_ virtually unchanged.^35^ At more negative potentials, however, *k*_*rec*_ rapidly drops with the applied potential, which is characteristic of a band-edge pinning (BEP) regime wherein the applied potential drops at the space charge region leading to steady increase of the band bending. The *k*_*tran*_ vs. the applied potential plot also displays two distinct regimes bounded by the crossover potential. Moving toward negative potentials, *k*_*tran*_ steadily increases until reaching a plateau. The increase of *k*_*tran*_ with applied potential is a sign of FLP, whereas BEP dominates if *k*_*tran*_ remains unchanged. In fact, in the FLP regime, the charging of the interface will shift the conduction and valence band edges causing the activation energy of the reaction (and thus *k*_*tran*_) to change with the applied potential.^35^ Alternatively, in the BEP regime, the conduction band will not change with the applied potential, and hence, *k*_*tran*_ will remain unaltered. These results provide compelling evidence of the existence of surface energy traps that extend about 1000 mV from the *V*_*fb*_ within the band gap causing the FLP. Note that the turn-on voltage, that is, the voltage at which the photocurrent starts to steadily increase with the applied potential, is close to the crossover potential (Figure 1b). This supports the observation that the photocurrent remains small at potentials more positive than the crossover point. In fact, recent studies on PEC water reduction using CIGS photocathodes revealed the existence of FLP, which appeared to be caused by energy traps that extended about 600 mV into the band gap from the valence band edge.^19^ It is likely that these energy traps, induced by surface vacancies of In and Ga, are responsible for the FLP observed during the PEC CO_2_ reduction.

Finally, we can use the IMPS data to compare the relative magnitudes of the rate constants for the HER (in aqueous electrolyte^19^) and the CO_2_-to-CO conversion. We note that although the rate constants obtained by IMPS are phenomenological, a qualitative comparison could be drawn given that the electrodes and experimental parameters are maintained and only the solvent and the reaction are altered. As expected, *k*_*tran*_(CO_2_) is about 1 order of magnitude lower than *k*_*tran*_(HER), which is consistent with the lower overpotential of the HER and the much larger availability of protons in water than CO_2_ in the M-ACN. This result also supports the assumption that the stability in AM likely originates from the faster depletion of surface photogenerated electrons.

In conclusion, bare CIGS photocathodes, without overlayers or cocatalysts, have demonstrated PEC CO_2_ reduction to CO at 1 Sun photocurrent densities commensurate with state-of-the-art PEC CO_2_RR systems using cocatalysts (Figure S15). Importantly, the performance is largely influenced by the electrolyte proton availability as shown by examining three representative solvents, namely an aprotic solvent with (M-ACN) and without (ACN) a proton donor and a protic solvent (AM). Adding a proton donor not only increased the partial photocurrent for CO as well as the selectivity but also reduced photocorrosion. However, in AM, the HER dominated over the CO_2_ reduction, likely due to the large excess of protons compared to CO_2_ molecules available and the fact that both compete for the same adsorption sites. Supported by PEIS data, which showed a significant drop in surface state concentration in M-ACN compared to ACN, it was suggested that the presence of protons activates an alternative reaction pathway (proceeding via a stabilized intermediate) to afford an accelerated charge transfer. The characterization of the charge carrier dynamics by IMPS revealed the occurrence of FLP in a wide potential range extending about 1 V into the band gap from the valence band edge, which significantly limits the turn-on voltage. Moreover, the relative rate constant for CO_2_ reduction in M-ACN remains 1 order of magnitude below that of the HER in aqueous electrolytes. Overall, these findings support the feasibility of direct SCLJs to drive the PEC CO_2_ reduction, although further studies are needed to suppress the FLP and the competing HER reaction.

## Supporting Information Available

Experimental details, SEM, AFM, EDX, Raman, XRD, IPCE, product quantification, EIS analysis, IMPS analysis, and comparison among state-of-the-art photocathodes for PEC CO_2_ reduction in organic solvent (PDF)

## References

[ref1] Navarro-Jaén S.; Virginie M.; Bonin J.; Robert M.; Wojcieszak R.; Khodakov A. Y. Highlights and Challenges in the Selective Reduction of Carbon Dioxide to Methanol. Nat. Rev. Chem. 2021, 5 (8), 564–579. 10.1038/s41570-021-00289-y. 37117584

[ref2] Wagner A.; Sahm C. D.; Reisner E. Towards Molecular Understanding of Local Chemical Environment Effects in Electro- and Photocatalytic CO_2_ Reduction. Nat. Catal. 2020, 3 (10), 775–786. 10.1038/s41929-020-00512-x.

[ref3] He J.; Janáky C. Recent Advances in Solar-Driven Carbon Dioxide Conversion: Expectations versus Reality. ACS Energy Lett. 2020, 5 (6), 1996–2014. 10.1021/acsenergylett.0c00645. 32566753 PMC7296618

[ref4] Li X.; Yu J.; Jaroniec M.; Chen X. Cocatalysts for Selective Photoreduction of CO_2_ into Solar Fuels. Chem. Rev. 2019, 119 (6), 3962–4179. 10.1021/acs.chemrev.8b00400. 30763077

[ref5] Jin S. What Else Can Photoelectrochemical Solar Energy Conversion Do Besides Water Splitting and CO_2_ Reduction?. ACS Energy Lett. 2018, 3 (10), 2610–2612. 10.1021/acsenergylett.8b01800.

[ref6] Chang X.; Wang T.; Yang P.; Zhang G.; Gong J. The Development of Cocatalysts for Photoelectrochemical CO_2_ Reduction. Adv. Mater. 2019, 31 (31), 1804710 10.1002/adma.201804710. 30537099

[ref7] Lai Y.; Watkins N. B.; Muzzillo C.; Richter M.; Kan K.; Zhou L.; Haber J. A.; Zakutayev A.; Peters J. C.; Agapie T.; Gregoire J. M. Molecular Coatings Improve the Selectivity and Durability of CO_2_ Reduction Chalcogenide Photocathodes. ACS Energy Lett. 2022, 7 (3), 1195–1201. 10.1021/acsenergylett.1c02762.

[ref8] Leung J. J.; Warnan J.; Ly K. H.; Heidary N.; Nam D. H.; Kuehnel M. F.; Reisner E. Solar-Driven Reduction of Aqueous CO_2_ with a Cobalt Bis(Terpyridine)-Based Photocathode. Nat. Catal. 2019, 2 (4), 354–365. 10.1038/s41929-019-0254-2.

[ref9] Deng X.; Li R.; Wu S.; Wang L.; Hu J.; Ma J.; Jiang W.; Zhang N.; Zheng X.; Gao C.; Wang L.; Zhang Q.; Zhu J.; Xiong Y. Metal–Organic Framework Coating Enhances the Performance of Cu_2_O in Photoelectrochemical CO_2_ Reduction. J. Am. Chem. Soc. 2019, 141, 10924–10929. 10.1021/jacs.9b06239. 31200598

[ref10] Hu Z.; Gong J.; Ye Z.; Liu Y.; Xiao X.; Yu J. C. Cu(In,Ga)Se_2_ for Selective and Efficient Photoelectrochemical Conversion of CO_2_ into CO. J. Catal. 2020, 384, 88–95. 10.1016/j.jcat.2020.02.015.

[ref11] Ikeda S.; Fujikawa S.; Harada T.; Nguyen T. H.; Nakanishi S.; Takayama T.; Iwase A.; Kudo A. Photocathode Characteristics of a Spray-Deposited Cu_2_ZnGeS_4_ Thin Film for CO_2_ Reduction in a CO_2_-Saturated Aqueous Solution. ACS Appl. Energy Mater. 2019, 2 (9), 6911–6918. 10.1021/acsaem.9b01418.

[ref12] Wu J.; Huang Y.; Ye W.; Li Y. CO_2_ Reduction: From the Electrochemical to Photochemical Approach. Adv. Sci. 2017, 4 (11), 1700194 10.1002/advs.201700194. PMC5700640 29201614

[ref13] Chi L.-P.; Niu Z.-Z.; Zhang X.-L.; Yang P.-P.; Liao J.; Gao F.-Y.; Wu Z.-Z.; Tang K.-B.; Gao M.-R. Stabilizing Indium Sulfide for CO_2_ Electroreduction to Formate at High Rate by Zinc Incorporation. Nat. Commun. 2021, 12 (1), 5835 10.1038/s41467-021-26124-y. 34611149 PMC8492718

[ref14] Shinagawa T.; Larrazábal G. O.; Martín A. J.; Krumeich F.; Pérez-Ramírez J. Sulfur-Modified Copper Catalysts for the Electrochemical Reduction of Carbon Dioxide to Formate. ACS Catal. 2018, 8 (2), 837–844. 10.1021/acscatal.7b03161.

[ref15] Wang K.; Ma Y.; Liu Y.; Qiu W.; Wang Q.; Yang X.; Liu M.; Qiu X.; Li W.; Li J. Insights into the Development of Cu-Based Photocathodes for Carbon Dioxide (CO_2_) Conversion. Green Chem. 2021, 23 (9), 3207–3240. 10.1039/D0GC04417B.

[ref16] Gu J.; Wuttig A.; Krizan J. W.; Hu Y.; Detweiler Z. M.; Cava R. J.; Bocarsly A. B. Mg-Doped CuFeO_2_ Photocathodes for Photoelectrochemical Reduction of Carbon Dioxide. J. Phys. Chem. C 2013, 117 (24), 12415–12422. 10.1021/jp402007z.

[ref17] Iwase A.; Yoshino S.; Takayama T.; Ng Y. H.; Amal R.; Kudo A. Water Splitting and CO_2_ Reduction under Visible Light Irradiation Using Z-Scheme Systems Consisting of Metal Sulfides, CoO_x_-Loaded BiVO_4_, and a Reduced Graphene Oxide Electron Mediator. J. Am. Chem. Soc. 2016, 138 (32), 10260–10264. 10.1021/jacs.6b05304. 27459021

[ref18] Yoshida T.; Yamaguchi A.; Umezawa N.; Miyauchi M. Photocatalytic CO_2_ Reduction Using a Pristine Cu_2_ZnSnS_4_ Film Electrode under Visible Light Irradiation. J. Phys. Chem. C 2018, 122 (38), 21695–21702. 10.1021/acs.jpcc.8b04241.

[ref19] Liu Y.; Bouri M.; Yao L.; Xia M.; Mensi M.; Grätzel M.; Sivula K.; Aschauer U.; Guijarro N. Identifying Reactive Sites and Surface Traps in Chalcopyrite Photocathodes. Angew. Chem., Int. Ed. 2021, 60 (44), 23651–23655. 10.1002/anie.202108994. PMC8597141 34428331

[ref20] Guijarro N.; Prévot M. S.; Yu X.; Jeanbourquin X. A.; Bornoz P.; Bourée W.; Johnson M.; Le Formal F.; Sivula K. A Bottom-Up Approach toward All-Solution-Processed High-Efficiency Cu(In,Ga)S_2_ Photocathodes for Solar Water Splitting. Adv. Energy Mater. 2016, 6 (7), 1501949 10.1002/aenm.201501949.

[ref21] White J. L.; Baruch M. F.; Pander J. E. III; Hu Y.; Fortmeyer I. C.; Park J. E.; Zhang T.; Liao K.; Gu J.; Yan Y.; Shaw T. W.; Abelev E.; Bocarsly A. B. Light-Driven Heterogeneous Reduction of Carbon Dioxide: Photocatalysts and Photoelectrodes. Chem. Rev. 2015, 115 (23), 12888–12935. 10.1021/acs.chemrev.5b00370. 26444652

[ref22] Piontek S.; junge Puring K.; Siegmund D.; Smialkowski M.; Sinev I.; Tetzlaff D.; Roldan Cuenya B.; Apfel U.-P. Bio-Inspired Design: Bulk Iron–Nickel Sulfide Allows for Efficient Solvent-Dependent CO_2_ Reduction. Chem. Sci. 2019, 10 (4), 1075–1081. 10.1039/C8SC03555E. 30774904 PMC6346401

[ref23] Moura de Salles Pupo M.; Kortlever R. Electrolyte Effects on the Electrochemical Reduction of CO_2_. ChemPhysChem 2019, 20 (22), 2926–2935. 10.1002/cphc.201900680. 31600018 PMC6899813

[ref24] Yoneyama H.; Sugimura K.; Kuwabata S. Effects of Electrolytes on the Photoelectrochemical Reduction of Carbon Dioxide at Illuminated P-Type Cadmium Telluride and p-Type Indium Phosphide Electrodes in Aqueous Solutions. J. Electroanal. Chem. Interfacial Electrochem. 1988, 249 (1), 143–153. 10.1016/0022-0728(88)80355-3.

[ref25] Zhao Q.; Martirez J. M. P.; Carter E. A. Revisiting Understanding of Electrochemical CO_2_ Reduction on Cu(111): Competing Proton-Coupled Electron Transfer Reaction Mechanisms Revealed by Embedded Correlated Wavefunction Theory. J. Am. Chem. Soc. 2021, 143 (16), 6152–6164. 10.1021/jacs.1c00880. 33851840

[ref26] Mendieta-Reyes N. E.; Cheuquepán W.; Rodes A.; Gómez R. Spectroelectrochemical Study of CO_2_ Reduction on TiO_2_ Electrodes in Acetonitrile. ACS Catal. 2020, 10 (1), 103–113. 10.1021/acscatal.9b02932.

[ref27] Rudnev A. V.; Zhumaev U. E.; Kuzume A.; Vesztergom S.; Furrer J.; Broekmann P.; Wandlowski T. The Promoting Effect of Water on the Electroreduction of CO_2_ in Acetonitrile. Electrochim. Acta 2016, 189, 38–44. 10.1016/j.electacta.2015.12.088.

[ref28] Hori Y.; Wakebe H.; Tsukamoto T.; Koga O. Electrocatalytic Process of CO Selectivity in Electrochemical Reduction of CO_2_ at Metal Electrodes in Aqueous Media. Electrochim. Acta 1994, 39 (11), 1833–1839. 10.1016/0013-4686(94)85172-7.

[ref29] Luo S.; Zeng L.; Xu D.; Kathe M.; Chung E.; Deshpande N.; Qin L.; Majumder A.; Hsieh T.-L.; Tong A.; Sun Z.; Fan L.-S. Shale Gas-to-Syngas Chemical Looping Process for Stable Shale Gas Conversion to High Purity Syngas with a H_2_:CO Ratio of 2:1. Energy Environ. Sci. 2014, 7 (12), 4104–4117. 10.1039/C4EE02892A.

[ref30] Liu Y.; Xia M.; Yao L.; Mensi M.; Ren D.; Grätzel M.; Sivula K.; Guijarro N. Spectroelectrochemical and Chemical Evidence of Surface Passivation at Zinc Ferrite (ZnFe_2_O_4_) Photoanodes for Solar Water Oxidation. Adv. Funct. Mater. 2021, 31 (16), 2010081 10.1002/adfm.202010081.

[ref31] Liu Y.; Le Formal F.; Boudoire F.; Guijarro N. Hematite Photoanodes for Solar Water Splitting: A Detailed Spectroelectrochemical Analysis on the PH-Dependent Performance. ACS Appl. Energy Mater. 2019, 2 (9), 6825–6833. 10.1021/acsaem.9b01261.

[ref32] Costentin C.; Drouet S.; Robert M.; Savéant J.-M. A Local Proton Source Enhances CO_2_ Electroreduction to CO by a Molecular Fe Catalyst. Science 2012, 338 (6103), 90–94. 10.1126/science.1224581. 23042890

[ref33] Wang F.; Wang J.; Cheng Y. Enhanced Photoreduction CO_2_ Efficiency by Criss-Crossed TiO_2_ Nanoflakes Combined with CdS under Visible Light. R. Soc. Open Sci. 2019, 6 (3), 181789 10.1098/rsos.181789. 31032034 PMC6458405

[ref34] Wang Y.; Wang H.; Woldu A. R.; Zhang X.; He T. Optimization of Charge Behavior in Nanoporous CuBi_2_O_4_ Photocathode for Photoelectrochemical Reduction of CO_2_. Catal. Today 2019, 335, 388–394. 10.1016/j.cattod.2018.12.047.

[ref35] Peter L. M. Energetics and Kinetics of Light-Driven Oxygen Evolution at Semiconductor Electrodes: The Example of Hematite. J. Solid State Electrochem. 2013, 17 (2), 315–326. 10.1007/s10008-012-1957-3.

