# Supplementary material for: Photoelectrochemical CO2 Reduction at a Direct CuInGaS2/Electrolyte Junction
Source: ACS Energy Lett. 2023 Mar 2;8(4):1645–51. doi: 10.1021/acsenergylett.3c00022 (PMC10111408; doi:10.1021/acsenergylett.3c00022)
Supplement: Supplementary file 1 — nz3c00022_si_001.pdf [file nz3c00022_si_001.pdf]

Supporting Information

Photoelectrochemical CO_2 Reduction at a Direct $CuInGaS_2$/Electrolyte Junction

Yongpeng Liu,[§,†] Meng Xia,[§] Dan Ren, Simon Nussbaum, Jun-Ho Yum, Michael Grätzel, Néstor Guijarro,[*,‡] Kevin Sivula*

Institute of Chemical Sciences and Engineering, École Polytechnique Fédérale de Lausanne (EPFL), Station 6, 1015 Lausanne, Switzerland

Corresponding Authors

E-mail: nestor.guijarro@ua.es
E-mail: kevin.sivula@epfl.ch

Present Addresses

†Yusuf Hamied Department of Chemistry, University of Cambridge, Lensfield Road, Cambridge, CB2 1EW, United Kingdom.
‡Institute of Electrochemistry, Universidad de Alicante, Apartat 99, E-03080 Alacant, Spain

Author Contributions

§Y. Liu and M. Xia contributed equally to this work.

Contents

S1 Methods S4

S1.1 Preparation of CIGS Photocathodes S4

S1.2 Preparation of Electrolytes S5

S1.3 Surface Roughness Factor Determination S5

S1.4 Photoelectrochemical Characterizations S5

S1.5 Intensity-Modulated Photocurrent Spectroscopy (IMPS) S6

S1.6 Gas Chromatography Measurements and Product Quantification S7

S2 Scanning Electron Microscope (SEM) Images S9

S3 Atomic Force Microscopy (AFM) Image S10

S4 Energy-Dispersive X-ray Spectroscopy (EDX) Mapping S11

S5 Raman Spectra and X-ray Diffraction (XRD) Pattern S12

S6 IPCE Measurements S13

S7 Control Experiments for CH_3OH Oxidation S14

S8 Control Experiments for CO Origination S16

S9 Electrochemical Impedance Spectroscopy Analysis S17

S10 Butler Plot S19

S11 Band Diagram. S20

S12 Intensity-Modulated Photocurrent Spectroscopy (IMPS) Analysis S21

S13 Comparison among State of the Art CO_2 Reduction Photocathodes in Organic Solvent S23

References S25

List of Figures

S1 Schematic of a cappuccino-type electrochemical cell S6

S2 Representative gas chromatography (GC) trace of PEC CO_2 reduction in M-ACN S8

S3 Scanning electron microscope (SEM) images S9

S4 Atomic force microscopy (AFM) image S10

S5 Energy-dispersive X-ray spectroscopy (EDX) mapping S11

S6 Raman spectra and XRD pattern S12

S7 IPCE measurements S13

S8 Gas chromatography (GC) trace of CH_3OH oxidation S15

S9 Control experiments for CO origination S16

S10 Nyquist plot of impedance response in M-ACN S17

S11 Butler plot S19

S12 Band diagram S20

S13 Nyquist plot of IMPS response S21

S14 Charge transfer efficiency (TE) S22

S15 Radar plot S23

List of Tables

S1 Product quantification for CH_3OH oxidation. S15

S2 List of state of the art photocathodes for PEC CO_2 reduction in organic solvent. S24

S1 Methods

S1.1 Preparation of CIGS Photocathodes

CIGS photocathodes were prepared by a solution-based colloidal method as described elsewhere.[S1] Briefly, 1 mmol copper(II) acetylacetonate ($Cu(CH_3COCHCOCH_3)_2$, 99.99+%, Sigma-Aldrich), 0.3 mmol indium(III) acetylacetonate ($In(CH_3COCHCOCH_3)_3$, 98%, Strem Chemicals), 0.7 mmol gallium(III) acetylacetonate ($Ga(CH_3COCHCOCH_3)_3$, 99.99%, Sigma-Aldrich) and 2 mmol sulfur (99.98%, Sigma-Aldrich) were mixed with 12 mL oleylamine (technical grade, 70%, Sigma-Aldrich) in a three necked flask. The mixture was degassed and dissolved by stirring at 130 °C for 30 min under vacuum. Subsequently, the solution precursor was then purged with Argon and heated up to 265 °C for 60 min to complete the reaction. After naturally cooling down the flask, the as-prepared colloidal CIGS nanocrystals were then purified. First, ethanol was added to the mixture and centrifuged to separate the nanocrystals pellet from the supernatant. Second, the nanocrystals were further washed by redispersing them in toluene, adding ethanol again to precipitate them and centrifuging the mixture to separate them. This procedure was repeated 3 times. Finally, the nanocrystal pellet was redispersed in 1-hexanethiol ($CH_3(CH_2)_5SH$, 95%, Sigma-Aldrich) to prepare the nanocrystal ink (200 mg mL^{-1}). The nanocrystal ink was tape-casted onto flexible molybdenum foils (0.1 mm, 99.95%, chemPUR) and annealed at 250 °C for 4 min in air in a hot plate (VWR 10027-246). This procedure was repeated twice to reach a film thickness of around 800 nm. The films were spray-coated with a 6.5 M $SbCl_3$ (99.9%, Aldrich) methanol solution (0.35 mmol antimony cm^{-2}, geometric area of electrode), flame-sealed in an ampoule containing 6 mg of sulfur powder and annealed in an oven pre-heated at 550 °C for 30 min. Finally, the ampoules were removed from the oven and let cool down naturally.

S1.2 Preparation of Electrolytes

3 types of electrolytes were used in electrochemical measurements, that are anhydrous acetonitrile (ACN), 1M CH_3OH in ACN (M-ACN), and anhydrous methanol (AM). In all the cases, 0.1 M Bu_4NPF_6 (Tetra-n-butylammonium hexafluorophosphate, 97%, fluorochem) was added as supporting electrolyte. ACN was taken from the acetonitrile bottle (99.9%, extra dry over molecular sieve, AcroSeal®, Acros). AM was taken from the methanol bottle ($\geq$ 99.9%, Fisher Chemical). M-ACN represents 1 M AM added into ACNe. All electrolytes were freshly prepared before the electrochemical measurements. The electrolytes were either saturated with CO_2 (99.998%, Carbagas) or Ar (99.998%, Carbagas) by sparging the electrolytes for 20 min.

S1.3 Surface Roughness Factor Determination

The roughness factor of CIGS film was determined by the atomic force microscopy (AFM). An average roughness factor of 1.22±0.02 was obtained by measuring 5 different samples.

S1.4 Photoelectrochemical Characterizations

Photoelectrochemical measurements were carried out in a cappuccino-type electrochemical cell (0.238 cm^2 active geometric area, Figure S1, full description can be found in[S2]) with a 3-electrode configuration: a CIGS working electrode, a Pt mesh counter electrode and a Ag/Ag^+ (acetonitrile/0.1 M tetrabutylammonium perchlorate/0.01 M $AgNO_3$) non-aqueous reference electrode (RE-7, ALS Co., Ltd). The 3-electrode configuration was controlled by a Bio-Logic SP-300 potentiostat. The applied potential was calibrated to ferrocene redox couple (Fc^+/Fc, 98%, Acros). A 450 W xenon arc lamp (Newport 66921), that was calibrated to AM 1.5G, was used as light source. Linear sweep voltammetry (LSV) was recorded at a scan rate of 20 mV/s. The incident photon-to-current efficiency (IPCE) was measured on a tunable light source platform (TLS-300XU, Newport) including a 300 W xenon arc lamp

(Newport 6258), a DC arc lamp power supply (OPS-A500 DC), a cornerstone 130 monochromator (CS130-USB-3-FH) and a research arc lamp housing (Newport 66902). The photon flux was calibrated by a calibrated Si photodiode (FDS100-CAL, Thorlabs). Impedance measurements were carried out on the Bio-Logic SP-300 potentiostat with frequency ranges from 5 MHz to 0.2 Hz and a 25 mV sinusoidal amplitude. Impedance data were fitted with equivalent circuits using modeling software ZView (Scribner Associates).

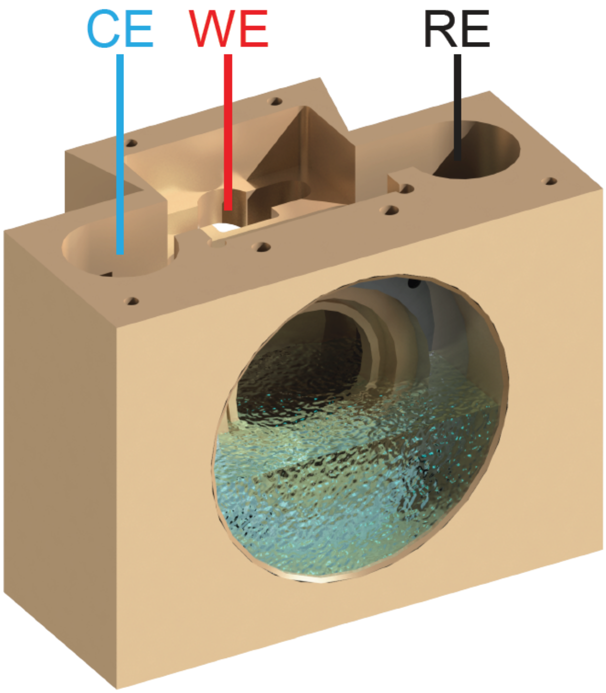

Figure S1: Schematic of a cappuccino-type electrochemical cell.

S1.5 Intensity-Modulated Photocurrent Spectroscopy (IMPS)

An array of white LED (Cree XLamp MC-E Color), that was powered by a background DC current of 600 mA, was used as the light source. An arbitrary function generator (Tektronix AFG3021C) was used to sinusoidally-modulate the light intensity in a frequency range of 50 kHz to 0.5 Hz with a 100 mA modulation amplitude (ca. 16% modulation depth). The applied potential on working electrode was controlled by a Keithley 2450 source measure unit (SMU) and the photocurrent response was monitored by a digital phosphor oscillo-

scope (Tektronix DPO7254C) through a differential probe (Tektronix TDP3500) in parallel with a 50 Ohm resistor (Velleman ED/E12) which was in series with the counter electrode. The entire IMPS setup was enclosed with blackout materials (Thorlabs TB4). IMPS data were fitted using custom Python program with lmfit package to perform curve fitting with nonlinear regression.

S1.6 Gas Chromatography Measurements and Product Quantification

Products quantification of the PEC CO_2 reduction on prepared CIGS photocathodes were performed in a homemade single-chamber PEEK cell using a CIGS photocathode as working electrode and a Pt foil as counter electrode, and a Ag/Ag^+ (acetonitrile/0.1 M tetrabutylammonium perchlorate/0.01 M $AgNO_3$) non-aqueous reference electrode (RE-7, ALS Co., Ltd). LCS-100 solar simulator (Newport, with air mass 1.5 G filter) was used as the light source. The light intensity was controlled with a calibrated silicon diode with KG 3 filter, by adjusting its distance from the light source. Before each test, CO_2 gas (99.999%, Carbagas) was infused into electrolyte for 10 min to saturate the electrolyte (6 mL) and it was continuously bubbled at a flow rate of 10 cm^3/min during the tests.

Chronoamperometry CO_2 reduction were performed under simulated one sun illumination at selected potentials by a Gamry potentiostat (Interface 1000). The gas products were periodically injected to and detected by an online gas chromatography (GC, Trace ULRTA, Thermo), where a micropacked shincarbon column (Restek) and pulse discharge detector (PDD, Vici) was used for gas separation and detection, separately. A standard gas purchased from Carbagas was used to calibrate the PDD peak signal, which was a mixture of multiple gaseous products (H_2, CO, CH_4, C_2H_4, and C_2H_6) of known concentration with CO_2 matrix. A representative GC trance of H_2 and CO in M-ACN is shown in Figure S2.

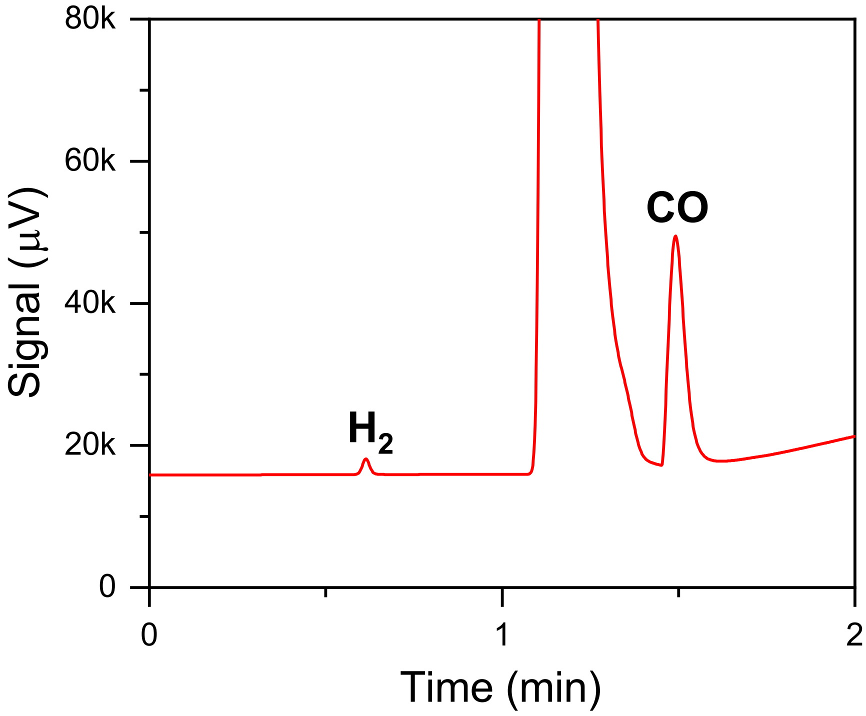

Figure S2: Representative gas chromatography (GC) trace of H_2 and CO from pulse discharge detector channel on CIGS photocathode measured at −1.86 V *vs.* Fc^+/Fc in M-ACN.

The Faradaic efficiency (FE) for producing each gas product (H_2 and CO) was calculated by the following equation:

$$FE(H_2) = \frac{2 \times n(H_2) \times N_A}{\frac{j_{t_0} \times \Delta t}{q}}$$

$$FE(CO) = \frac{2 \times n(CO) \times N_A}{\frac{j_{t_0} \times \Delta t}{q}}$$

where n(H_2) and n(CO) is the amount of H_2 and CO detected in one GC injection (mol), N_A is the Avogadro constant, j_{t_0} is the photocurrent during the injection, Δt is the time required to fill the sample loop (100 μL) of GC, and q is the elementary charge.

S2 Scanning Electron Microscope (SEM) Images

Scanning electron microscope (SEM) configurations (Zeiss Gemini) for these images were 3 kV electron high tension (EHT), 150 pA probe current, 3 mm working distance, 20 μm aperture size and In-Lens annular secondary electrons detector. CIGS thin film photocathodes with thickness around 800 nm and average grain size of ca. 450 nm were fabricated by a solution based method as described in the Section S1.1.

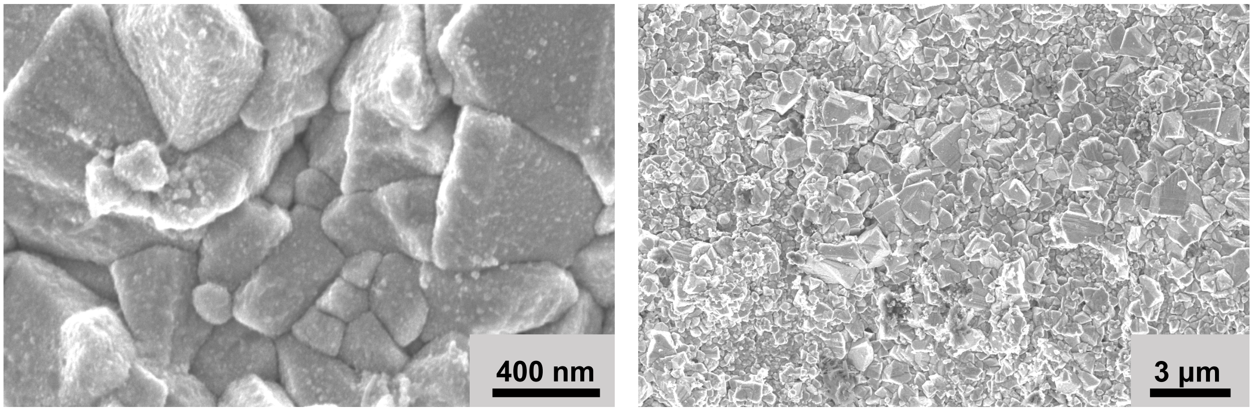

Figure S3: SEM images of CIGS films at different magnifications.

S3 Atomic Force Microscopy (AFM) Image

Topographic image was acquired on an Asylum Research Cypher S AFM.

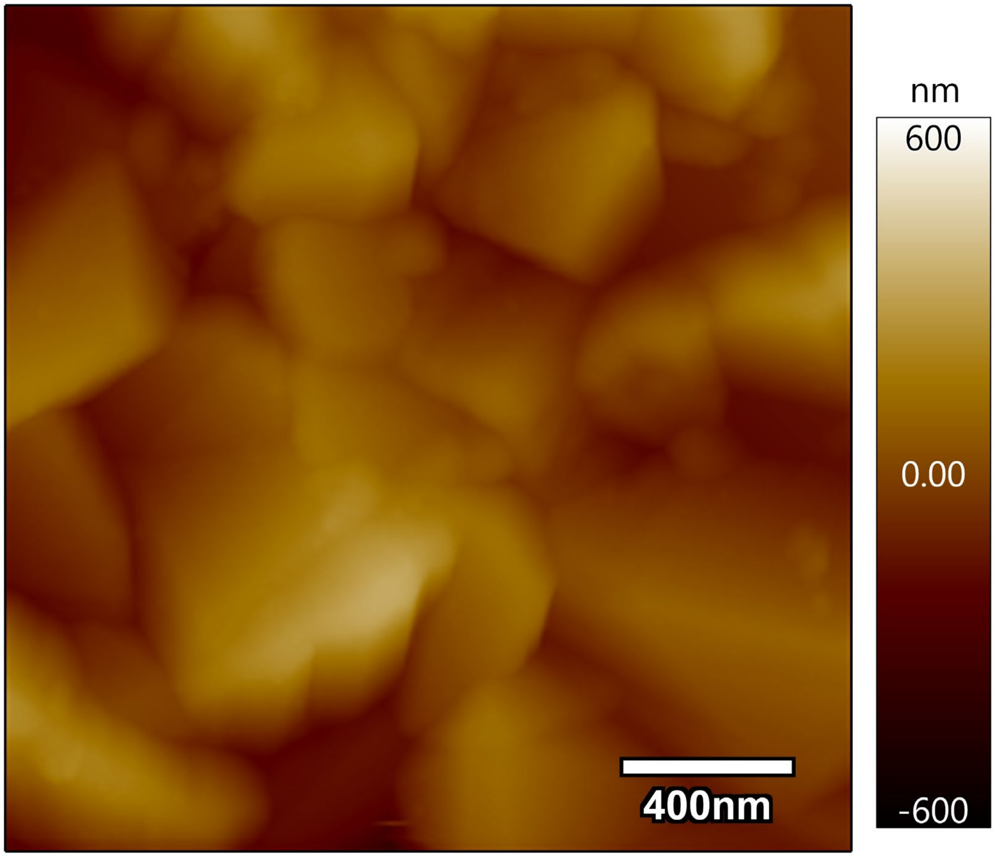

Figure S4: Topographic image of CIGS film.

S4 Energy-Dispersive X-ray Spectroscopy (EDX) Mapping

Energy-dispersive X-ray spectroscopy (EDX) was performed on a 20 mm^2 silicon drift detector (Oxford Instruments X-Max^N 80) with the following SEM configurations: 10 kV EHT, 800 pA probe current, 8.5 mm working distance, 30 μm aperture size and SE2 Everhart-Thornley secondary electrons detector. EDX mapping and quantification were performed on the AZtec software (Oxford Instruments). Based on 5 different EDX quantification, the stoichiometric numbers of Cu, In, Ga, S are: 1±0.01, 0.26±0.02, 0.69±0.01, 2.2±0.04. Representative EDX spectrum and mapping of $CuIn_{0.3}Ga_{0.7}S_2$ films are shown in Figure S5. Note that the EDX analysis corroborates that the bulk and fed stoichiometry match well.

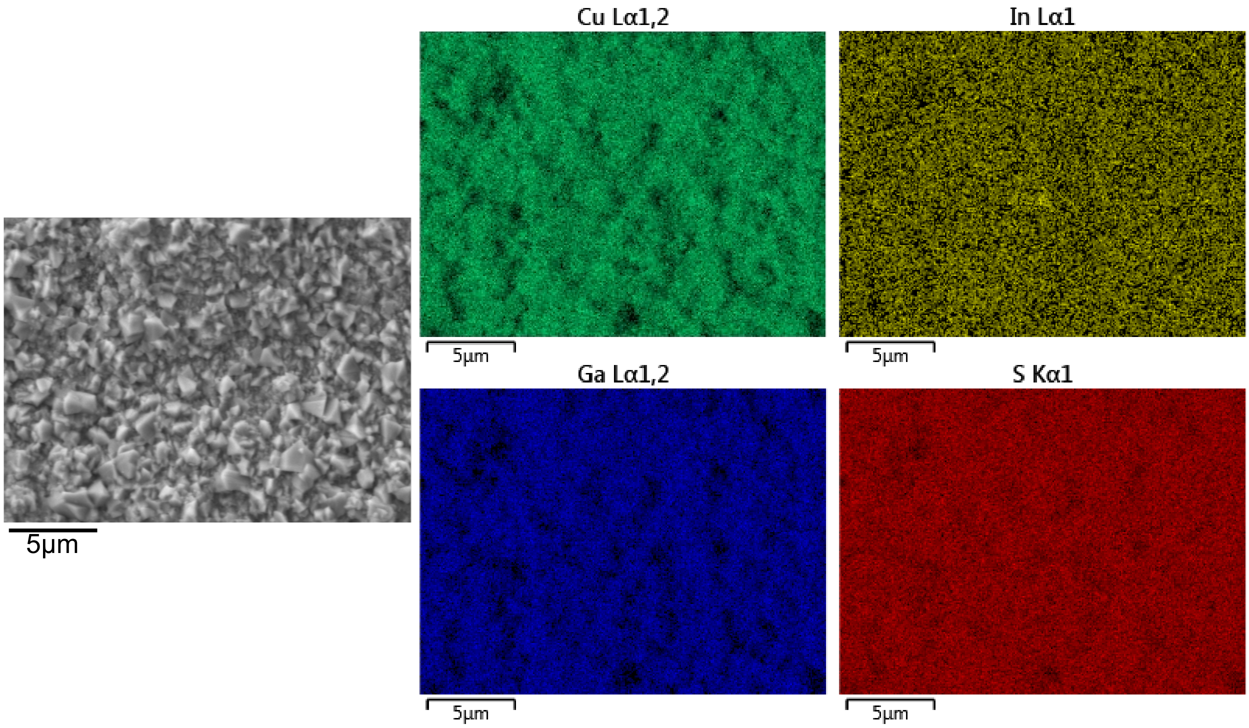

Figure S5: Representative EDX mapping for CIGS films.

S5 Raman Spectra and X-ray Diffraction (XRD) Pattern

Raman spectra were acquired by a confocal Raman microscope (HORIBA Jobin Yvon XploRA PLUS) containing an optical microscope (Olympus BX41), a 532 nm laser (HORIBA DPSS) and a charge-coupled device (CCD) camera (HORIBA Jobin Yvon Syncerity).

X-ray diffraction (XRD) pattern was recorded on a Bruker D8 Discover diffractometer with a non-monochromatized Cu-source, a Nickel filter and a LYNXEYE XE energy-dispersive 1-D detector in Bragg-Brentano geometry.

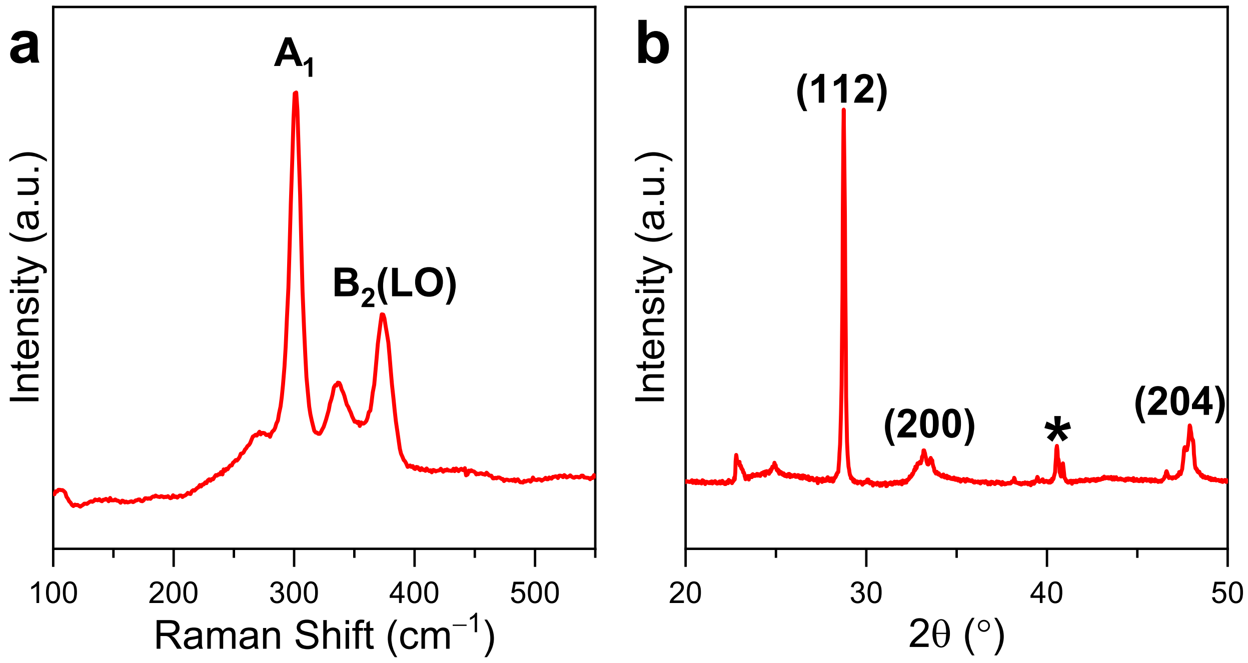

Figure S6: Representative (a) Raman spectra and (b) XRD pattern for CIGS films. A star (*) denotes XRD peaks associated to the substrate (Mo and MoS_2). References for peak assignments.[S1,S3]

S6 IPCE Measurements

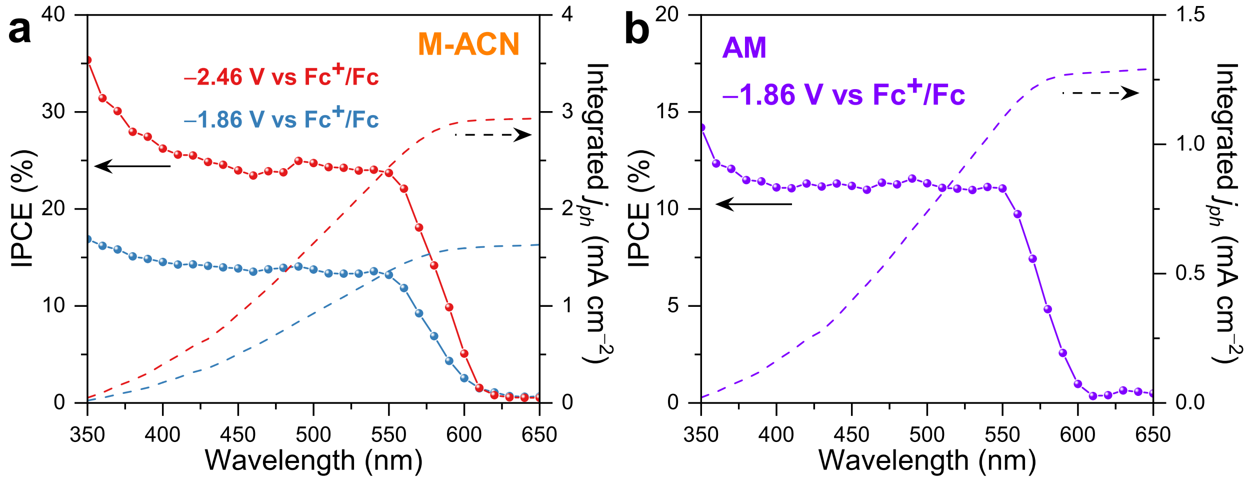

Figure S7: IPCE measurements in (a) M-ACN and (b) AM.

S7 Control Experiments for CH_3OH Oxidation

CH_3OH has been widely used as hole scavenger for photocatalytic water splitting. Under certain circumstances, H_2 could be one by-product due to CH_3OH oxidation.[S4] Likewise, CH_3OH oxidation has been extensively studied in direct methanol fuel cells as the anode reaction where the CH_3OH-to-CO_2 conversion is the dominant oxidation reaction.[S5] It is essential to validate if CH_3OH oxidation has any contribution towards the H_2 we observed during PEC CO_2 reduction. With the aim of resolving the origination of produced H_2, we performed chronoamperometry on two Pt foils at 4 mA/cm^2 (-2.1 V *vs.* Fc^+/Fc) in pure CH_3OH with 0.1 M TBAP in the same gas tight cell with He as the carrier gas to simulate PEC CO_2 reduction condition on CIGS photocathodes. Considering H_2 production is dominated by proton reduction on the cathode, there are two potential scenarios in such control experiments. Scenario 1 - CH_3OH oxidation produces H_2, the Faradaic efficiency (FE) of H_2 should be greater than 100 % because both cathodic and anodic current are contributing to H_2 production. Scenario 2 - CH_3OH oxidation produces only CO_2, the FE of H_2 should be close to 100 % because of proton reduction and we should observe the peak of CO_2 in gas chromatography (GC).

GC trace of CH_3OH oxidation is displayed in Figure S8 where both H_2 and CO_2 are clearly observed. The control experiments have been performed twice and product quantification results are list in Table S1. As we observed, FE of H_2 is close to 100 % and CO_2 peak signal is significant (quantification of CO_2 is not available due to the absence of calibration gas). This is a sign that our experimental condition is close to scenario 2, similar to the case of direct menthol fuel cells, where the dominate oxidation reaction is CH_3OH-to-CO_2 conversion. Note that we observe trace of CO which is an intermediate during CH_3OH oxidation in a direct menthol fuel cell.[S6]

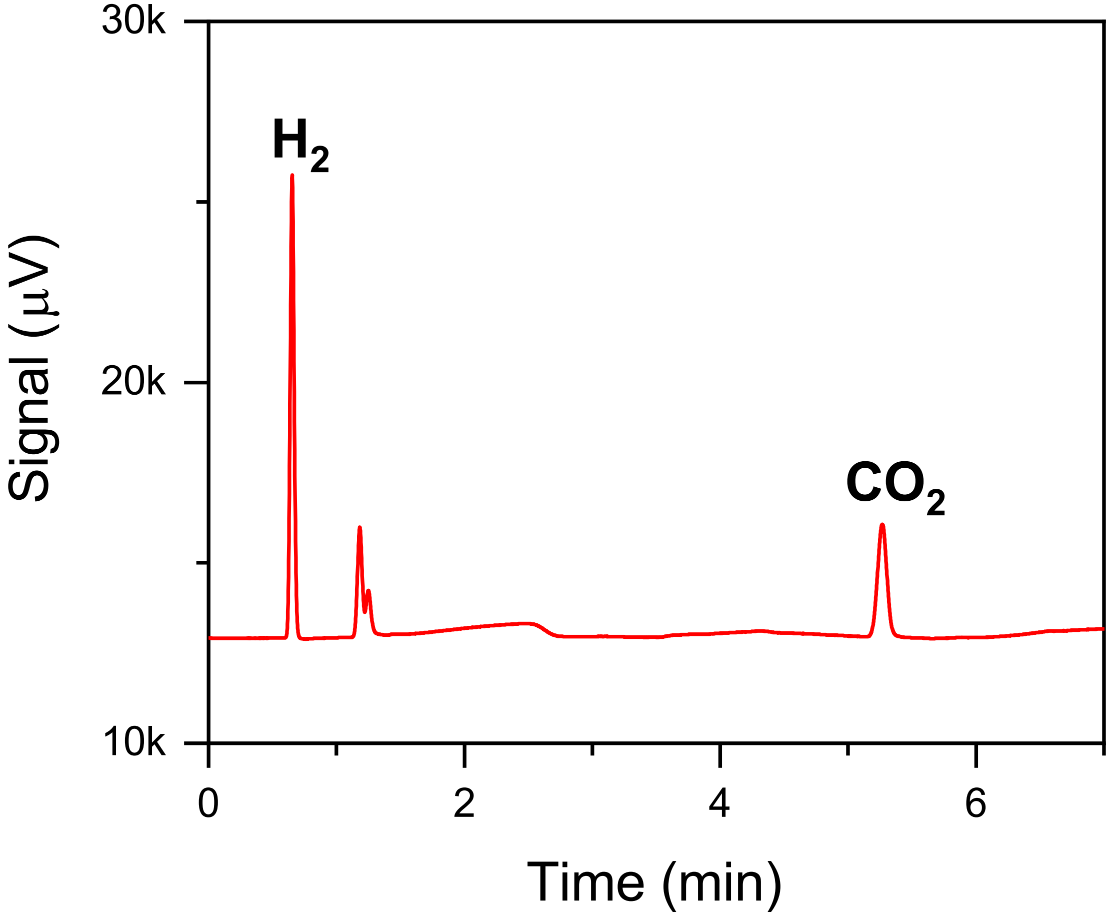

Figure S8: Gas chromatography (GC) trace of CH_3OH oxidation on two Pt foils measured at 4 mA/cm^2 (-2.1 V *vs.* Fc^+/Fc) in pure CH_3OH with 0.1 M TBAP.

Table S1: Product quantification for CH_3OH oxidation.

H_2 (%)	CO (%)	CO_2 (%)
99.64	0.08	NA
99.87	0.09	NA

S8 Control Experiments for CO Origination

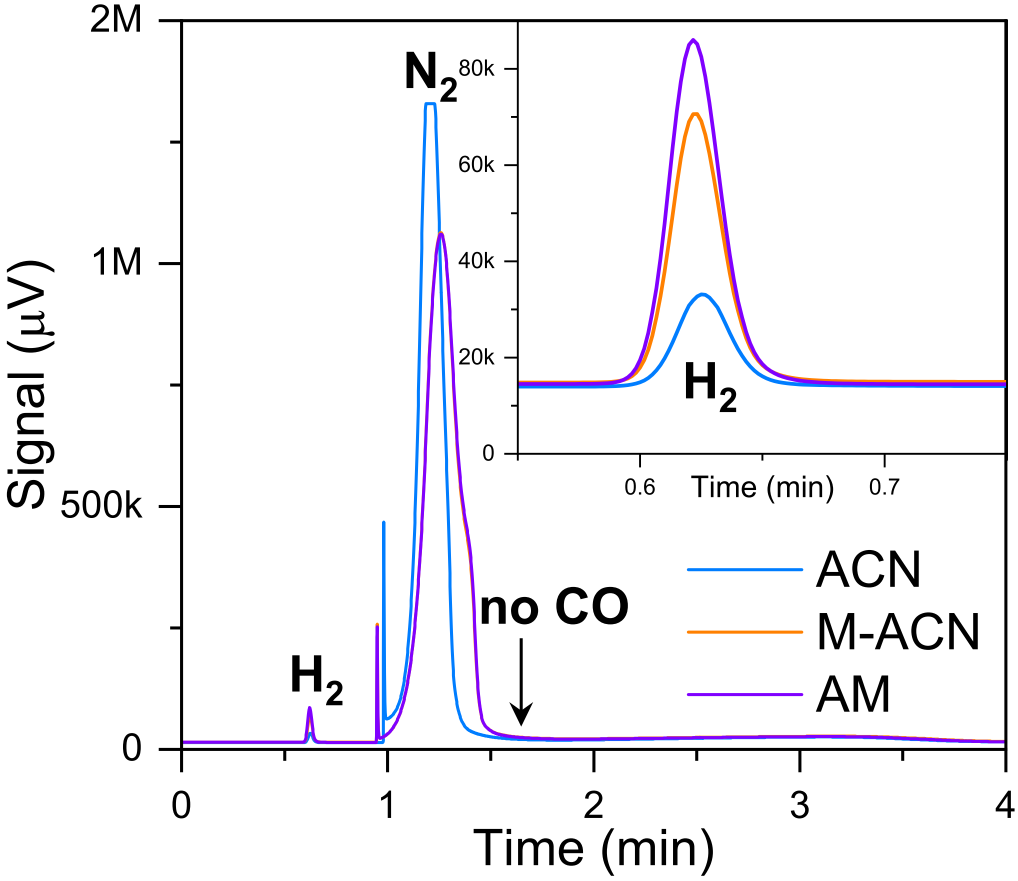

Figure S9: Gas chromatography (GC) trace of control experiments performed under PEC conditions in N_2-saturated electrolytes.

S9 Electrochemical Impedance Spectroscopy Analysis

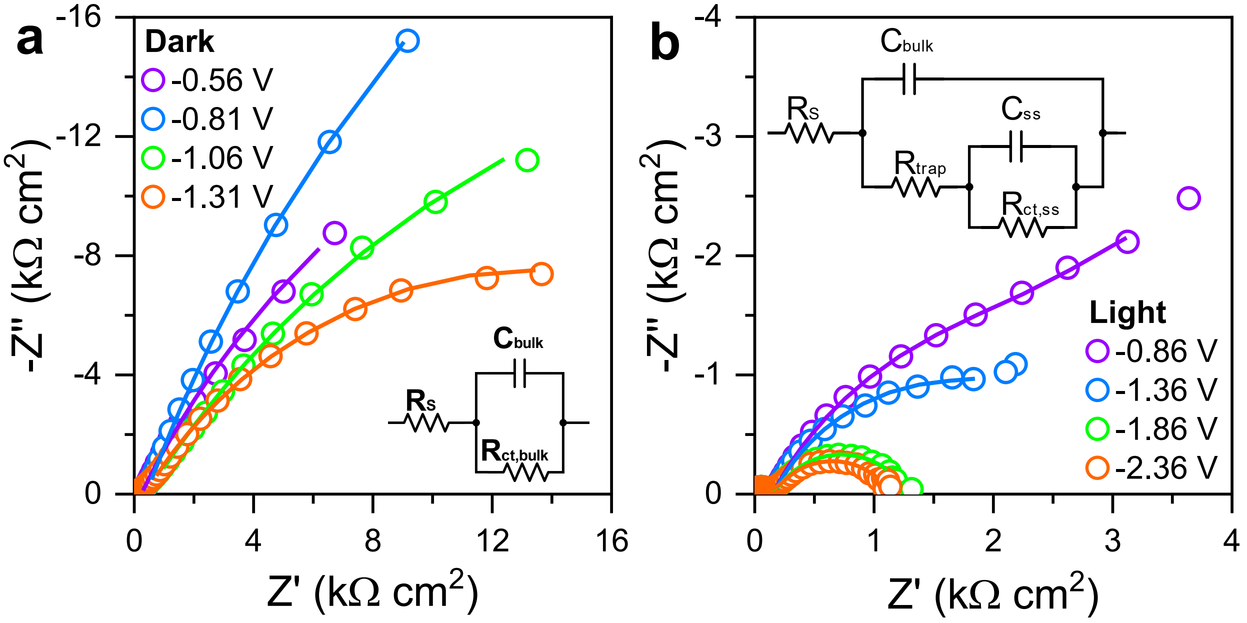

Figure S10: Nyquist plot of impedance response (open circle) measured (a) in the dark and (b) under illumination in M-ACN at 4 representative voltage with corresponding fitting curves (solid line). Equivalent circuits employed in the fitting are displayed in the figure as inset.

Mott–Schottky (MS) analysis (Figure 4a) based on the value of C_{bulk} is performed in dark condition in Figure S10a:

$$C_{bulk}^{-2} = \frac{2}{q\varepsilon\varepsilon_0 A^2 N_A}(V - V_{fb} - \frac{kT}{q}) \tag{1}$$

where q is elementary charge, ε is relative permittivity (taking 10 for $CuIn_{0.3}Ga_{0.7}S_2$),[S7] ε_0 is vacuum permittivity, A is the effective surface area that results from multiplying the geometric area by the surface roughness factor (1.22±0.02), N_A is acceptor density, V is applied potential, V_{fb} is flat-band potential, k is Boltzmann constant and T is absolute temperature.

Mott–Schottky analysis reveals a V_{fb} of −0.5 V *vs.* Fc^+/Fc and a N_A of 1.68×10^{18} cm^{-3}.

Density of surface states ($DOSS$) is converted from the value of C_{SS} when EIS is performed under illumination in Figure S10b:

$$DOSS = \frac{C_{SS}}{q} \tag{2}$$

Total density of surface states (N_{SS}) is determined by integrating the $DOSS$ as a function of voltage:

$$N_{SS} = \int_V DOSS(V)\ dV \tag{3}$$

S10 Butler Plot

The flat-band potential (V_{fb}) has also been estimated by the Butler method,[S8] where a V_{fb} of −0.504 V *vs.* Fc^+/Fc has been obtained from Figure S11.

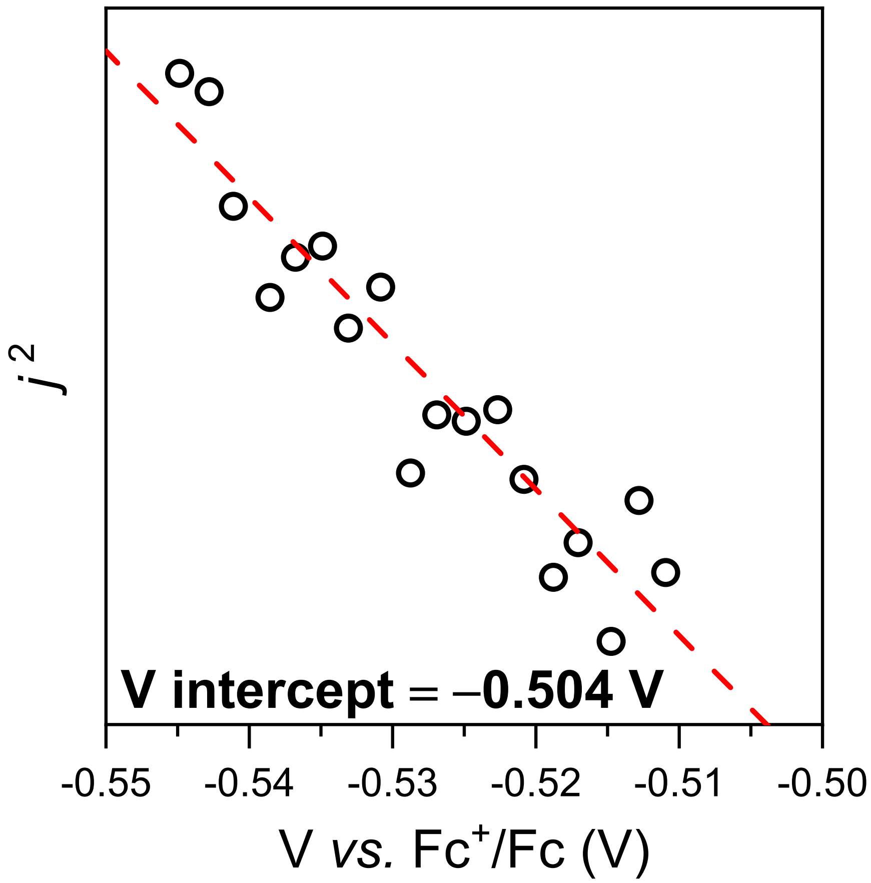

Figure S11: Butler plot.

S11 Band Diagram

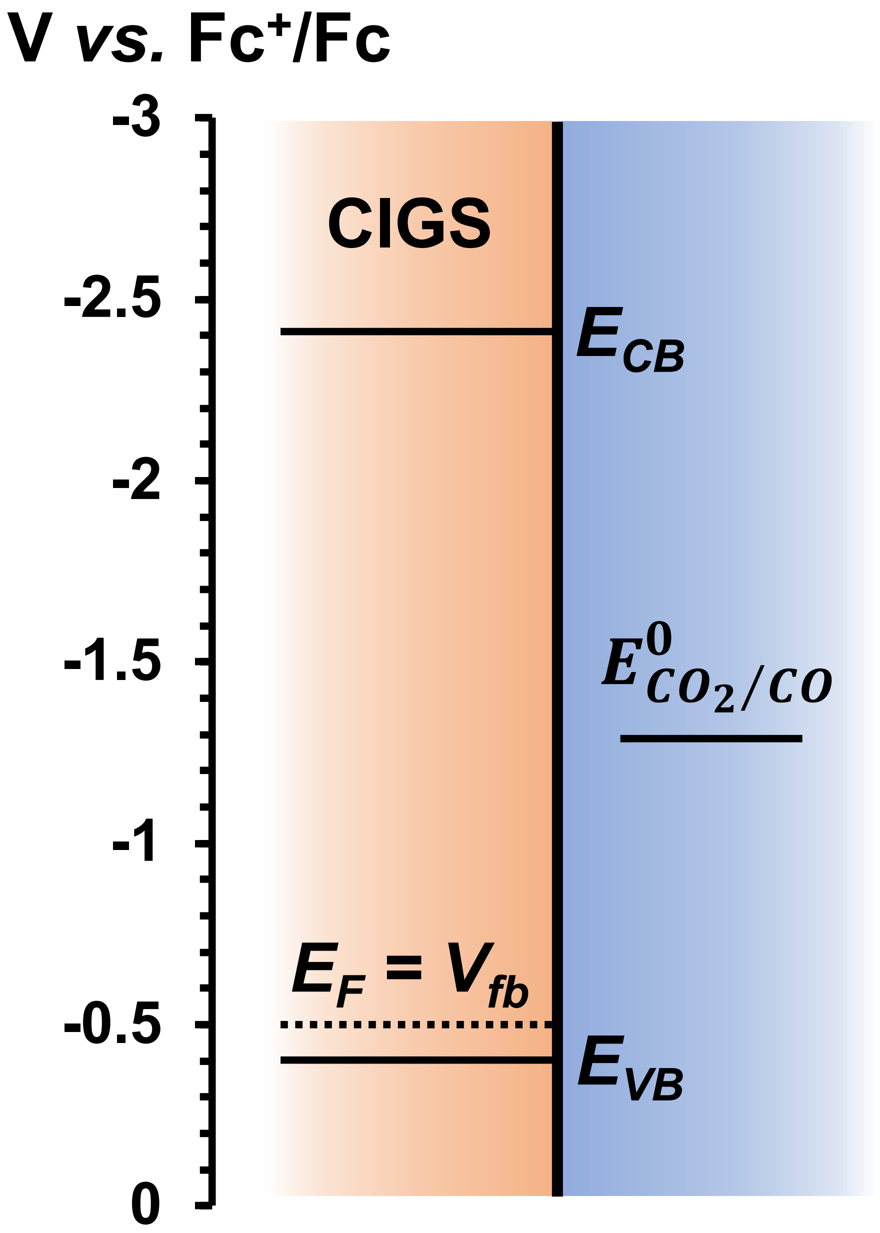

Figure S12: Band diagram of CIGS at flat band condition. $E^0_{CO_2/CO}$ represents the redox potential of CO_2/CO in acetonitrile, which is around −1.28 V *vs.* Fc^+/Fc.[S9,S10]

S12 Intensity-Modulated Photocurrent Spectroscopy (IMPS) Analysis

Intensity-modulated photocurrent response $j(\omega)$ is fit to the following equation:[S11]

$$j(\omega) = j_e \times \frac{k_{tran} + i\omega(\frac{C_H}{1+C_H/C_{SC}})}{k_{tran} + k_{rec} + i\omega} \times \frac{1}{1 + (i\omega\tau_d)^{\alpha}} \qquad (4)$$

where j_e represents the electron current towards interface and τ_d is the mean transit time for photogenerated holes. k_{tran} and k_{rec} are pseudo-first order rate constant for charge transfer and recombination, respectively. C_{SC} and C_H represent capacitance of the space charge layer and the Helmholtz layer. α ($0< \alpha \leq 1$) is introduced as a non-ideality factor.

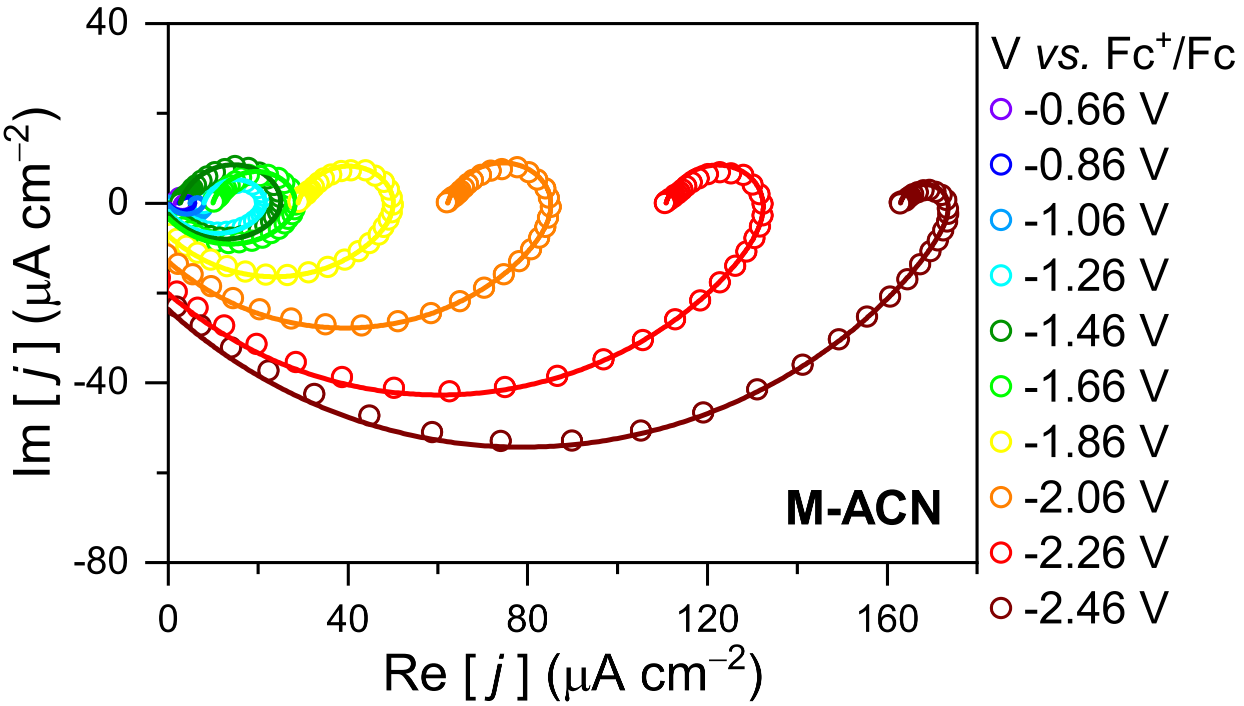

Figure S13: Nyquist plot of IMPS response (open circle) measured at 10 different voltages in M-ACN with the corresponding fitting curves (solid line).

The charge transfer efficiency (TE) is determined by the following equation and plotted as a function of potential in Figure S14:

$$TE(\%) = \frac{k_{tran}}{k_{tran} + k_{rec}} \times 100\% \tag{5}$$

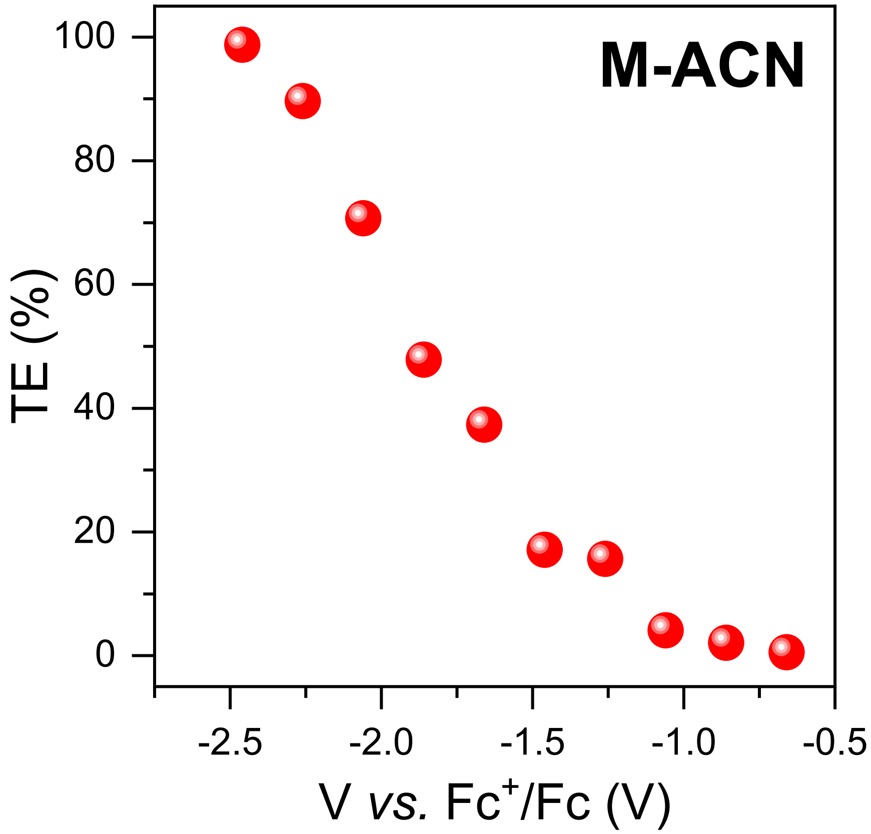

Figure S14: Charge transfer efficiency (TE) as a function of potential in M-ACN.

As we observed, the trend of TE follows the development of j_{ph} in Figure 1b and the distribution of $DOSS$ (Figure 4a) has effects on TE in the potential range from -1.0 V to -1.5 V *vs.* Fc^+/Fc.

S13 Comparison among State of the Art CO_2 Reduction Photocathodes in Organic Solvent

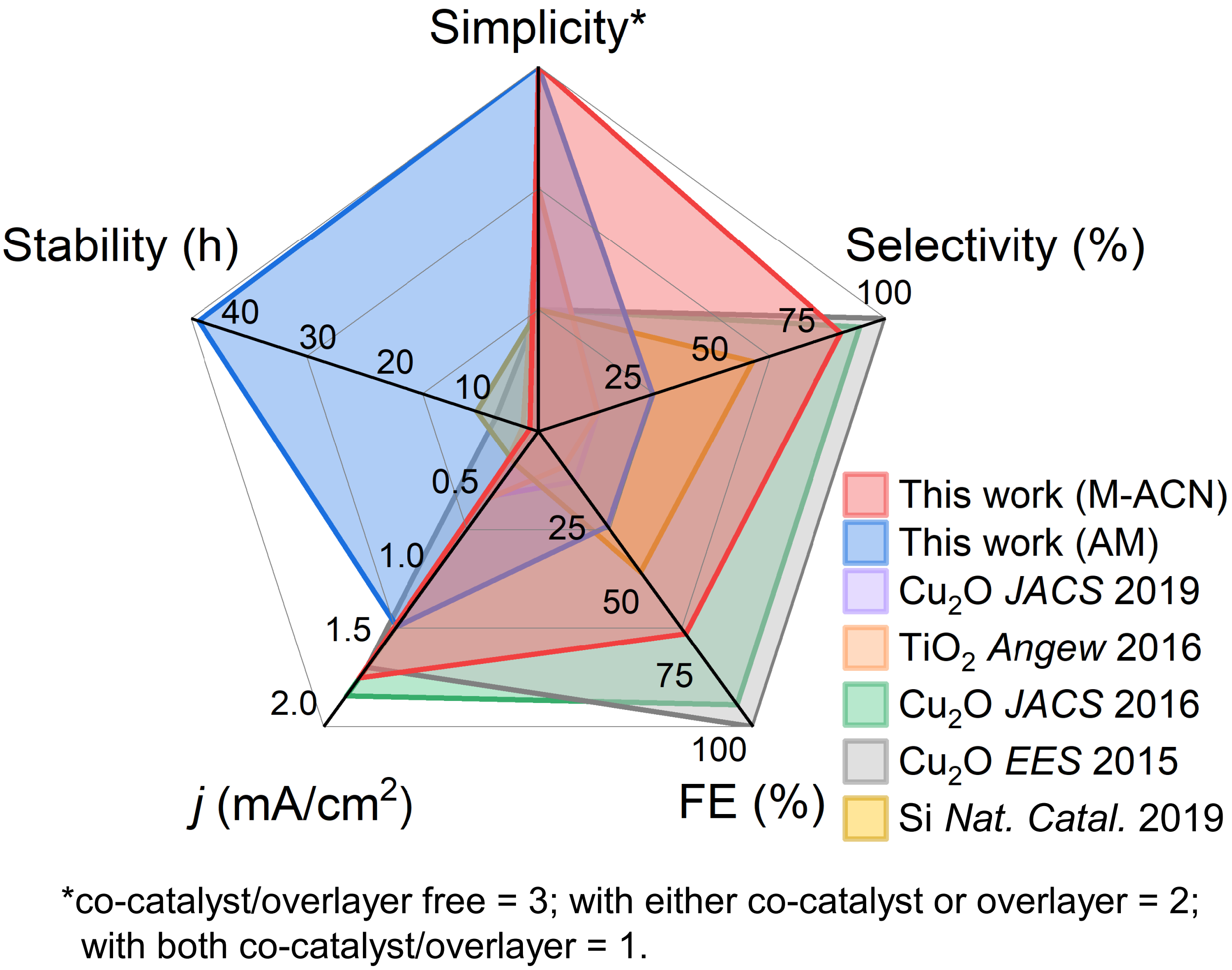

Figure S15: Comparison of device simplicity, selectivity, Faradaic efficiency (FE), photocurrent density (j) and stability against representative photocathodes for PEC CO_2 reduction in organic solvent. Detailed comparison can be found in Table S2.

Table S2: List of state of the art photocathodes for PEC CO_2 reduction in organic solvent.

Photocathode	Co-catalyst	Electrolyte	Illumination condition	Photocurrent /Potential	Product (FE)	Stability (remaining photocurrent)	Reference
$CuInGaS_2$	none	0.1 M Bu_4NPF_6 and 1 M CH_3OH in CH_3CN	450 W Xenon lamp (AM 1.5G)	1.67 mA/cm^2 /–1.86 V vs Fc$^+$/Fc	CO (68.6%) H_2 (11%)	1.1 h (0.99 mA/cm^2)	This work (M-ACN)
$CuInGaS_2$	none	0.1 M Bu_4NPF_6 in CH_3OH	450 W Xenon lamp (AM 1.5G)	1.32 mA/cm^2 /–1.86 V vs Fc$^+$/Fc	CO (32.1%) H_2 (65.6%)	44 h (no sign of degradation)	This work (AM)
Cu_2O	$Cu_3(BTC)_2$	0.1 M Bu_4NPF_6 in CH_3CN	300 W Xenon lamp (AM 1.5G)	0.44 mA/cm^2 / –2.27 V vs Fc$^+$/Fc	CO (17%)	400 s / 0.4 mA/cm^2 / –1.97 V vs Fc$^+$/Fc (no sign of degradation)	*J. Am. Chem. Soc.* **2019**, 141, 10924−10929
Au/Cu_2O/Al:ZnO/TiO_2	Re(bipy–tBu)$(CO)_3$Cl	0.1 M Bu_4NPF_6 in CH_3CN	450 W Xenon lamp (AM 1.5G)	1.79 mA/cm^2 / –1.9 V vs Fc$^+$/Fc	CO (85-100%)	1.05 h (0.25 mA/cm^2)	*J. Am. Chem. Soc.* **2016**, 138, 1938−1946
Au/Cu_2O/Al:ZnO/TiO_2	2 mM Re(tBu-bipy)$(CO)_3$Cl	0.1 M Bu_4NPF_6 and 7.5 M CH_3OH in CH_3CN	450 W Xenon lamp (AM 1.5G)	1.6 mA/cm^2 / –1.73 V vs Fc$^+$/Fc	CO (100%)	5.5 h (1.5 mA/cm^2)	*Energy Environ. Sci.* **2015**, 8, 855–861
TiO_2	33.6 nmol fac-[MnBr(4,4′-(PO_3Et_2)bpy)$(CO)_3$] on 1.0 cm^2 TiO_2	0.1 M Bu_4NBF_4 in CH_3CN/H_2O (19/1)	Solar simulator (100 mW/cm^2, λ>420 nm)	0.45 mA (area not specified) / –1.7 V vs Fc$^+$/Fc	CO (12%) H_2 (59%)	2 h (NA)	*Angew. Chem. Int. Ed.* **2016**, 55, 7388–7392
p-InP	none	0.3 M Bu_4NClO_4 in CH_3OH	500 W Xenon lamp (480 mW/cm^2)	50 mA/cm^2 / –2.1 V vs Ag QRE	CO (60%) H_2 (33%) CH_3COOH (16%)	NA (a visually observable white indium film is formed)	*J. Phys. Chem. B* **1998**, 102, 9834–9843; *J. Electrochem. Soc.* **1998**, 145, L82
p-InP	none	0.08 M LiOH in CH_3OH	5000 W Xenon lamp	3.5 mA/cm^2 / –2.3 V vs Ag/AgCl sat. KCl	CO (39%) H_2 (24%) HCOOH (15%)	NA	*Chem. Eng. J.* **2006**, 116, 227–231
p-CdTe	none	0.1 M Bu_4NClO_4 and 5% H_2O in DMF	450 W Xenon lamp (4.1 mW/cm^2, 600 nm monochromatic)	1.6 mA/cm^2 / –1.6 V vs SCE	CO (80.6%) H_2 (<0.3%)	NA	*Electrochimica Acta* **1984**, 29, 923–932
p-NiO/ZnTCPP	Re(dmbpy)	0.1 M Bu_4NPF_6 in DMF	500 W Xenon lamp (6 mW, 430 nm monochromatic)	110 μA/cm^2 / voltage not specified	CO (13%)	250 s (110 μA/cm^2)	*J. Catal.* **2014**, 310, 57–66
p-WSe_2	1 mM Re$(CO)_3$(dmbpy)Cl	0.1 M Bu_4NClO_4 in CH_3CN	5 mW He/Ne laser (22 mW/cm^2)	0.14 mA (area not specified) / –1.5 V vs SSCE	CO (predominant product with unit current efficiency)	NA	*J. Electroanal. Chem.* **1986**, 209, 101–107
p-GaAs	none	0.08 M LiOH in CH_3OH	5000 W Xenon lamp	3.4 mA/cm^2 / –2.4 V vs Ag/AgCl sat. KCl	CO (25%) H_2 (34%) HCOOH (15%)	NA	*Chem. Eng. J.* **2006**, 116, 227–231
p-GaAs	none	0.3 M Bu_4NClO_4 in CH_3OH	500 W Xenon lamp (480 mW/cm^2)	50 mA/cm^2 / –2.2 V vs Ag QRE	CO (11%) H_2 (76%) CH_3COOH (19%)	NA (small extent of surface corrosion)	*J. Phys. Chem. B* **1998**, 102, 9834–9843
p-Si/TiO_2	CotpyP	0.1 M Bu_4NBF_4 in CH_3CN/H_2O (6/4)	150 W solar simulator (100 mW/cm^2, λ>400 nm)	220 μA/cm^2 / –1.0 V vs Fc$^+$/Fc	CO (47.6%) H_2 (16.7%) HCOOH (12.8%)	8 h / 90 μA/cm^2 / –1.0 V vs Fc$^+$/Fc (no sign of degradation)	*Nat. Catal.* **2019**, 2, 354–365
p-Si	none	0.3 M Bu_4NClO_4 in CH_3OH	500 W Xenon lamp (480 mW/cm^2)	50 mA/cm^2 / –2.1 V vs Ag QRE	CO (17%) H_2 (82%) CH_3COOH (19%)	NA	*J. Phys. Chem. B* **1998**, 102, 9834–9843
p-Si	1 mM Re$(CO)_3$(dmbpy)Cl	0.1 M Bu_4NClO_4 in CH_3CN	5 mW He/Ne laser (22 mW/cm^2)	0.5 mA/cm^2 / –1.2 V vs SSCE	CO (predominant product with unit current efficiency)	NA	*J. Electroanal. Chem.* **1986**, 209, 101–107
p-Si	6 mM Re(bipy-Bu')$(CO)_3$Cl	0.1 M Bu_4NPF_6 in CH_3CN	661 nm laser diode (95 mW/cm^2)	31 mA/cm^2 / –1.75 V vs Ag/AgCl	CO (97±3%)	NA	*J. Phys. Chem. C* **2010**, 114, 14220–14223

References

(S1) Guijarro, N.; Prévot, M. S.; Yu, X.; Jeanbourquin, X. A.; Bornoz, P.; Bourée, W.; Johnson, M.; Le Formal, F.; Sivula, K. A Bottom-Up Approach toward All-Solution-Processed High-Efficiency $Cu(In,Ga)S_2$ Photocathodes for Solar Water Splitting. *Adv. Energy Mater.* **2016**, *6*, 1501949.

(S2) Lopes, T.; Andrade, L.; Ribeiro, H. A.; Mendes, A. Characterization of photoelectrochemical cells for water splitting by electrochemical impedance spectroscopy. *Int. J. Hydrog. Energy* **2010**, *35*, 11601–11608.

(S3) Liu, Y.; Bouri, M.; Yao, L.; Xia, M.; Mensi, M.; Grätzel, M.; Sivula, K.; Aschauer, U.; Guijarro, N. Identifying Reactive Sites and Surface Traps in Chalcopyrite Photocathodes. *Angew. Chem. Int. Ed.* **2021**, *60*, 23651–23655.

(S4) Zhao, J.; Shi, R.; Li, Z.; Zhou, C.; Zhang, T. How to make use of methanol in green catalytic hydrogen production? *Nano Select* **2020**, *1*, 12–29.

(S5) Yaqoob, L.; Noor, T.; Iqbal, N. Recent progress in development of efficient electrocatalyst for methanol oxidation reaction in direct methanol fuel cell. *Int. J. Energy Res.* **2021**, *45*, 6550–6583.

(S6) Chung, D. Y.; Kim, H.-i.; Chung, Y.-H.; Lee, M. J.; Yoo, S. J.; Bokare, A. D.; Choi, W.; Sung, Y.-E. Inhibition of CO poisoning on Pt catalyst coupled with the reduction of toxic hexavalent chromium in a dual-functional fuel cell. *Sci. Rep.* **2014**, *4*, 1–5.

(S7) Frick, J. J.; Cava, R. J.; Bocarsly, A. B. Chalcopyrite $CuIn(S_{1-x}Se_x)_2$ for Photoelectrocatalytic H_2 Evolution: Unraveling the Energetics and Complex Kinetics of Photogenerated Charge Transfer in the Semiconductor Bulk. *Chem. Mater.* **2018**, *30*, 4422–4431.

(S8) Butler, M. Photoelectrolysis and physical properties of the semiconducting electrode WO_2. *J. Appl. Phys.* **1977**, *48*, 1914–1920.

(S9) Costentin, C.; Drouet, S.; Robert, M.; Savéant, J.-M. A local proton source enhances CO_2 electroreduction to CO by a molecular Fe catalyst. *Science* **2012**, *338*, 90–94.

(S10) Schreier, M.; Gao, P.; Mayer, M. T.; Luo, J.; Moehl, T.; Nazeeruddin, M. K.; Tilley, S. D.; Grätzel, M. Efficient and selective carbon dioxide reduction on low cost protected Cu_2O photocathodes using a molecular catalyst. *Energy Environ. Sci.* **2015**, *8*, 855–861.

(S11) Ponomarev, E. A.; Peter, L. M. A generalized theory of intensity modulated photocurrent spectroscopy (IMPS). *J. Electroanal. Chem.* **1995**, *396*, 219–226.
